# Targeting KK-LC-1 inhibits malignant biological behaviors of triple-negative breast cancer

**DOI:** 10.1186/s12967-023-04030-9

**Published:** 2023-03-09

**Authors:** Xudong Zhu, Jiawen Bu, Tong zhu, Yi Jiang

**Affiliations:** 1 Department of Oncology, Shengjing Hospital of China Medical University, Shenyang, 110004 Liaoning People’s Republic of China; 2 Department of General Surgery, Cancer Hospital of China Medical University, Liaoning Cancer Hospital and Institute, Shenyang, 110042 Liaoning People’s Republic of China

**Keywords:** Breast cancer, KK-LC-1, MAL2, MUC1-C, Prognosis, Tumor metastasis, Targeted drug

## Abstract

**Background:**

Cancer/testis antigens (CTAs) participate in the regulation of malignant biological behaviors in breast cancer. However, the function and mechanism of KK-LC-1, a member of the CTA family, in breast cancer are still unclear.

**Methods:**

Bioinformatic tools, immunohistochemistry, and western blotting were utilized to detect the expression of KK-LC-1 in breast cancer and to explore the prognostic effect of KK-LC-1 expression in breast cancer patients. Cell function assays, animal assays, and next-generation sequencing were utilized to explore the function and mechanism of KK-LC-1 in the malignant biological behaviors of triple-negative breast cancer. Small molecular compounds targeting KK-LC-1 were also screened and drug susceptibility testing was performed.

**Results:**

KK-LC-1 was significantly highly expressed in triple-negative breast cancer tissues than in normal breast tissues. KK-LC-1 high expression was related to poor survival outcomes in patients with breast cancer. In vitro studies suggested that KK-LC-1 silencing can inhibit triple-negative breast cancer cell proliferation, invasion, migration, and scratch healing ability, increase cell apoptosis ratio, and arrest the cell cycle in the G0–G1 phase. In vivo studies have suggested that KK-LC-1 silencing decreases tumor weight and volume in nude mice. Results showed that KK-CL-1 can regulate the malignant biological behaviors of triple-negative breast cancer via the MAL2/MUC1-C/PI3K/AKT/mTOR pathway. The small-molecule compound Z839878730 had excellent KK-LC-1 targeting ability and cancer cell killing ability. The EC_50_ value was 9.7 μM for MDA-MB-231 cells and 13.67 µM for MDA-MB-468 cells. Besides, Z839878730 has little tumor-killing effect on human normal mammary epithelial cells MCF10A and can inhibit the malignant biological behaviors of triple-negative breast cancer cells by MAL2/MUC1-C/PI3K/AKT/mTOR pathway.

**Conclusions:**

Our findings suggest that KK-LC-1 may serve as a novel therapeutic target for triple-negative breast cancer. Z839878730, which targets KK-LC-1, presents a new path for breast cancer clinical treatment.

**Supplementary Information:**

The online version contains supplementary material available at 10.1186/s12967-023-04030-9.

## Background

Current data suggests that breast cancer has been a malignant tumor with the highest morbidity and second highest mortality among all kinds of cancers. The number of new cases of breast cancer accounts for approximately 30% of the total number of new malignant tumors in women annually, which is a great threat to women’s health [1, 2]. Although the establishment of breast cancer molecular subtypes and the emergence of comprehensive treatment have greatly improved the survival outcomes of breast cancer patients, triple-negative breast cancer has the characteristics of strong heterogeneity, high malignancy, and the worst prognosis compared with other molecular types. Currently, there are no effective clinical treatment plans [3–5]. Recurrence and metastasis are the leading causes of death in patients with triple-negative breast cancer [6, 7]. Therefore, to clarify the regulatory mechanisms of biological behaviors, such as invasion and metastasis of triple-negative breast cancer, screening new therapeutic targets and improving the treatment and survival outcomes of patients are the focus of triple-negative breast cancer research.

Kita-Kyushu lung cancer antigen-1 (KK-LC-1), also called CT83 or Cxorf61 [8], is a member of the CTAs protein family [9]. The CTA family of proteins are mainly expressed in tumors and testicular tissues, but display low to no expression in other normal tissues. Therefore, KK-LC-1 protein is only expressed in cancer and testis tissues. Studies regarding KK-LC-1 have mainly focused on gastric, lung, and liver cancer [10–13]. A previous study found KK-LC-1 expression was higher in early gastric cancer tissues. Therefore, it was a biomarker for early gastric cancer. KK-LC-1 expression is related to *Helicobacter pylori* infection [14]. In lung cancer, KK-LC-1 expression is related to lymph node metastasis. The co-expression of KK-LC-1 and CTAs MAGE-A4 and NY-ESO-1 is related to poor survival outcomes in non-small cell lung cancer patients [15, 16]. In liver cancer, increased KK-LC-1 expression caused by hypomethylated CpG islands can physiologically bind to presenilin-1 to modulate the activity of the Notch1/Hes1 pathway, further promoting the proliferation and migration of liver cancer cells, thereby promoting the progression of hepatocellular carcinoma [13].

Few studies have explored the role of KK-LC-1 in breast cancer. The present research found that *KK-LC-1* mRNA expression was higher in triple-negative breast cancer tissues than in normal tissues [17]. KK-LC-1 has also been identified as a potential target for T cell-based immunotherapy [18]. The results of bioinformatic analysis suggested that KK-LC-1 expression was molecular subtype-specific [9, 19, 20], and KK-LC-1 was mainly expressed in triple-negative breast cancer [21–23]. Herein, we explored the expression and prognostic effects of KK-LC-1 on patients with triple-negative breast cancer and the role of KK-LC-1 expression in the development of triple-negative breast cancer using in vitro and in vivo experiments. We further investigated the regulatory mechanism of KK-LC-1 in the malignant behavior of breast cancer cells. Finally, we screened for a small-molecule compound targeting KK-LC-1. We hope KK-LC-1 may serve as a new therapeutic target, and this study will present a new path for triple-negative breast cancer clinical treatment.

## Methods

### Breast cancer clinical specimens

This research involved three independent study cohorts. The first cohort included six pairs of fresh triple-negative breast cancer and adjacent cancer tissues obtained from the China Medical University affiliated hospital between January 2020 and June 2020. These six pairs of samples were used to detect KK-LC-1 expression using western blotting. The second cohort included 481 breast cancer operation specimens obtained from the China Medical University affiliated hospital between April 2006 and November 2014. These specimens were used to detect KK-LC-1 expression using immunohistochemistry. Patient information included age, T grade, N grade, menopausal status, date of operation, date of local recurrence and distant metastasis, date of death, and molecular subgroups. All specimens were pathologically verified to have invasive ductal carcinoma. None of the patients received radiotherapy or chemotherapy before the operation and had complete postoperative follow-up data. Disease-free survival (DFS) refers to the time from operation completion to recurrence or distant organ metastasis. Overall survival (OS) refers to the time from operation completion to the death of a patient. If the patient did not develop local recurrence, distant metastasis, or the patient did not die, the last follow-up time was the endpoint of survival.

The third cohort included 126 triple-negative breast cancer patient specimens obtained from the China Medical University affiliated hospital between October 2012 and December 2014. These specimens were utilized to detect the expression of KK-LC-1, MAL2, MUC1, and CLDN2 by immunohistochemistry. This study was approved by the China Medical University Ethics Committee.

### Bioinformation analysis

The Cancer Genome Atlas (TCGA) database was applied to analyze the expression of 276 types of CTAs in breast cancer and adjacent cancer tissues. The STRING database was applied to construct protein–protein interaction (PPI) networks. The Oncomine database was utilized to analyze CT83 expression in different subgroups of breast cancer and adjacent tissues. The CCLE database was utilized to determine the expression of *CT83* in different cell lines. The Sangerbox tool (http://www.sangerbox.com/tool) was utilized to analyze the expression of *CT83*, *MAL2*, and *MUC1* genes in breast cancer and adjacent tissues, and was used to perform single gene set enrichment analysis (GSEA) to further clarify the potential pathways of *CT83*, *MAL2*, and *MUC1* genes using next-generation sequencing (NGS) data. The TISIDB database was utilized to analyze the expression of *CT83* in different breast cancer molecular subtypes. The Kaplan–Meier plotter database was applied to analyze the prognostic effect of *CT83* and *MAL2* gene expression in breast cancer patients.

The GEPIA database was utilized to analyze the expression of *MAL2* and *MUC1* in breast cancer and adjacent tissues. It was also utilized to analyze the prognostic effect of *MAL2* gene expression in patients with breast cancer. The UALCAN database was utilized to analyze the expression of *MAL2* and *MUC1* in breast cancer and adjacent tissues, and to analyze the prognostic effect of *MAL2* gene expression in patients with breast cancer. The PrognoScan database was utilized to analyze the prognostic effect of *MAL2* and *MUC1* gene expression in breast cancer patients from certain Gene Expression Omnibus (GEO) datasets.

### Western blot

Cell or tissue lysates were separated by sodium dodecyl sulfate–polyacrylamide gel electrophoresis on 10% gels, and proteins were transferred to PVDF membranes. The PVDF membranes were blocked with 5% nonfat dry milk for 1.5 h. Diluted primary antibodies were then added. The samples were incubated overnight on a shaker at 4 °C. The next day, appropriate secondary antibodies (1:10,000) were added and incubated at room temperature for 1 h. A list of antibodies utilized in the study is available in Additional file 1.

### Immunohistochemical experiments

The embedded paraffin tissue was cut into 5 µm sections. Slices were soaked in three containers of xylene for 15 min in sequence. The slices were soaked in containers containing 100%, 95%, and 90% alcohol for 10 min, and then placed in containers containing 80% and 60% alcohol for 10 min. The slices were immersed in EDTA solution and microwaved at high temperature for 20 min. These cells were then washed with PBS. Thereafter, 100 µL BSA solution was added to each section and blocked at RT for 30 min. Diluted primary antibody was added to the sliced tissue and incubated at RT for 1.5 h. Thereafter the secondary antibody was added, and the cells were incubated at RT for 45 min. The prepared DAB solution was added to the sliced tissues and incubated for 5–10 min. The sections were counterstained with hematoxylin in a dropwise manner for 2–5 min, and placed in hydrochloric acid alcohol for 10–20 s. The slices were soaked in containers containing 70%, 80%, 90%, 95%, and 100% alcohol for 10 min, and then soaked in three containers containing xylene for 5 min.

### Analyzing experimental results of immunohistochemistry

The results of the immunohistochemical experiments were determined by staining intensity and proportion of positive cells. Tissue staining intensity was graded as follows: colorless, 0; pale yellow, 1; brownish yellow, 2; and tan, 3. The proportion of positive cells was graded as follows: < 5% of positive cells, 0; 5–25% of positive cells, 1; 25–50% of positive cells, 2; 50–75% of positive cells, 3; and > 75% proportion of positive cells, 4. The product of the staining intensity score and the score of positive cell ratio was the final score for the tissue. A score ≥ 4 was considered high expression and < 4 was considered low expression.

### Lentiviral transfection

Cells were digested, centrifuged, and plated in six-well plates at 5 × 10^5^ cells per well. Transfection was performed when cells reached 50–70% confluency. The co-transfection reagent and calculated amount of virus were added to a 6-well plate. The target sequence for *CT83* was 5′ GGCGAAATGTCATCAAATT 3′. The Negative Control (NC) RNA sequence was 5′-GCAGTGAAAGATGTAGCCAAA-3′.

### Plasmid transfection

Experiments were performed according to the standard of 4 µL lipo3000, 5 µL p3000, 2.5 µg MAL2 overexpression plasmid/KK-LC-1 overexpression plasmid/HNRNPL silencing plasmid, and 2.5 µg NC plasmid per well in a 6-well plate. Two EP tubes were prepared, plasmid and p3000 was added to one, and lipo3000 was added to the other. The contents were mixed well and incubated for 5 min at RT (MEM medium was pre-added to each EP tube). The liquids were then added to the two EP tubes, mixed well, and allowed to stand for 15 min. It was then added to a 6-well plate, and the medium was changed after 6 h to complete plasmid transfection.

### CCK-8 cell proliferation assay

The cells were digested, centrifuged, resuspended, counted, and seeded in 96-well cell culture plates. The density was 5000 cells/well. Each group consisted of three sub-wells. Cells were allowed to adhere and were then cultured in an incubator for 24, 48, and 72 h, respectively. CCK-8 reagent (10 µL) was added to the culture medium at the corresponding time points and incubated at 37 °C for 2 h. The absorbance value at 450 nm was detected using a microplate reader, and the tumor cell proliferation curve was drawn.

### Cell migration and invasion assay

To determine cell migration, 6 × 10^4^ cells were plated in each upper Transwell chamber. After incubation for 24 h, the chambers were removed and washed thrice with PBS. The cells in the upper layer of the chamber were gently wiped off with a cotton swab, and 600 µL of paraformaldehyde was added to the 24-well plate corresponding to the lower layer of the chamber and fixed for 30 min. After three washes with PBS, 600 µL of crystal violet staining solution was added and the cells were stained for 30 min. Images were captured using a microscope and the results were analyzed. To determine cell invasion, the Transwell chamber was placed in the corresponding position of the 24-well plate. Matrigel was diluted in a serum-free medium (medium: Matrigel = 9:1). Diluted Matrigel (100 µL) was added to the upper layer of each chamber. Thereafter, the 24-well plate with the chamber was placed in the incubator for 24 h, and the 24-well plate was removed. The subsequent methodology, microscopic photography, and analysis of results were the same as those of the cell migration experiment.

### Scratch healing assay

The cells were digested, centrifuged, resuspended, counted, and plated in 24-well plates at a density of 5 × 10^4^ cells per well. Then a 100 µL pipette tip was utilized to make a cross scratch in a 24-well plate, the suspended cells were washed with PBS. Cells were photographed by an inverted microscope. After 24 h, photographs were obtained from the same field of view. The scratch distance was measured using software to calculate cell mobility.

### Annexin V-APC/PI cell apoptosis assay

Cells were harvested using EDTA-free trypsinization, washed twice with sterile PBS at 2000 rpm for 5 min, and collected. Binding Buffer (500 µL) was added to resuspend the cells. Annexin V-APC (5 µL) and PI staining solution (5 µL) were added and mixed well, respectively. Cell suspensions were protected from light at room temperature and allowed to react for 5–15 min. Within 1 h, the flow cytometer was utilized to detect the results.

### Cell cycle assay

The cells were digested, centrifuged, and resuspended, and 5 × 10^5^ cells were collected. PBS (500 µL) was added to resuspend the cells. The prepared single-cell suspension was carefully transferred to 1.2 mL of cold absolute ethanol and cells were resuspended by pipetting. Cells were fixed by placing in a freezer at − 20 °C for 2 h. PBS was added to the fixed cell suspension and the cells were collected at 1000 rpm for 3 min. After discarding the supernatant, RNase A was added to fully suspend the cells. Thereafter, PI was added, mixed, and incubated in the dark at 4 °C for 30 min.

### NGS at the RNA level

We selected MDA-MB-231 KK-LC-1 silenced cells and control cells for NGS, with three replicates per group. Sequencing was completed by Beijing Huada Protein Research and Development Center Co., Ltd.

### RT-qPCR

Total RNA from samples were isolated using TRIzol solution (Solarbio Company). The extracted RNA was reverse-transcribed by a cDNA synthesis kit (TaKaRa). qPCR was performed by SYBR Green PCR Master Mix (TaKaRa) and primers which bind to the KK-LC-1, MAL2, MUC1, CLDN2 and β-actin. These primers were designed by Shanghai Sangon Biotech Co., Ltd.

(KK-LC-1: forward, 5′-GAGCAGCATTCTGTGTGCCT-3′ and reverse 5′-CCCGAGAGAGGTCGTAGACTG-3′;

MAL2: forward, 5′-TGCCTCCTCCAATGTTCCTCTACC-3′ and reverse 5′-AATTTGAGCCACCATGCCAGAGAG-3′;

MUC1: forward, 5′-CTGCTGGTGCTGGTCTGTGTTC-3′ and reverse 5′- GGGTACTCGCTCATAGGATGGTAGG-3′;

CLDN2: forward, 5′-GAATCCCGAGCCAAAGACAGAGTG-3′ and reverse 5′-CAGTGGTGAGTAGAAGTCCCGTAGG-3′;β-actin: Forward, 5ʹ-GGCTGTATTCCCCTCCATCG-3ʹ and Reverse, 5ʹ-CCAGTTGGTAACAATGCCATGT-3ʹ).

The cycling protocol was: 95 °C for 30 s, followed by 40 denaturation cycles at 95 °C for 3 s, and finally annealing and extension at 60 °C for 30 s. Relative mRNA levels were calculated using the 2^−ΔΔCt^ method.

### Establishment of tumorigenic model in nude mice

BALB/c immunodeficient nude mice were utilized in the study and were housed in specific-pathogen free conditions. A total of eight, 5–6-week-old female mice, with a body weight of 16–18 g, were included. Cells in the MDA-MB-231/NC and MDA-MB-231/KD (KD: KK-LC-1 knockdown) groups were digested, resuspended, counted, and 1 × 10^7^ cells in each group were subcutaneously inoculated into nude mice. A Vernier caliper was used to regularly measure the size of the tumor (long diameter (a) and short diameter (b)). After 21 days, the nude mice were sacrificed and the tumors were excised, weighed, and photographed.

### RNA binding protein immunoprecipitation (RIP) assay

Magna RIP kit was used in this assay according to the manufacturer’s protocols. Protein A/G Agarose beads were incubated with primary antibodies overnight at 4 °C. Transfected cells were incubated in RIP lysis buffer. Then, lysates were incubated with magnetic beads-antibody complexes at 4 °C. After a brief elution, the purified RNAs were detected by RT-qPCR.

### α-amanitin treatment

Half-life of CLDN2 and β-actin was measured in cells treated with 50 mM α-amanitin every 6 h by RT-qPCR. 18S rRNA served as internal control.

### Screening of small-molecule compounds

The screening of small-molecule compounds targeting KK-LC-1 was completed by the MCE Company. Based on the protein structure of KK-LC-1, MCE preliminarily screened the small-molecule compound Z839878730, which can target KK-LC-1 through its compound library. Z839878730 was dissolved using dimethyl sulfoxide (DMSO).

### Molecular docking assay to analyze the binding of Z839878730 small-molecule compound to the KK-LC-1 protein

AlphaFold 2 was used to predict the structure of KK-LC-1. Residues 1–29 and 53–105 in the structure were two α-helices, and the pLDDT scores were high. The remaining residues had no fixed secondary structure and the pLDDT scores were low, which may have indicated isolated unstructured regions. To optimize the structures predicted by AlphaFold 2, 100 ns molecular dynamics simulations were run to cluster the 100 ns trajectory structures using the DBSCAN clustering algorithm. The chemical structure of Z839878730 was downloaded from PubChem. In this study, LeDocksoftware (http://www.lephar.com/index.htm) was used for molecular docking. Owing to the small size of the protein, the binding site was predicted by blind docking, and 200 conformations were generated to ensure the search was as detailed as possible. Conformation space, clustering with RMSD = 1.0 Å, and other parameters were set by default. The docking results were used to select the conformation with the lowest binding energy, which was analyzed and plotted using PLIP and PyMOL.

### Cell viability inhibition assay with small-molecule compounds

Breast cancer cells (5000 per well) were inoculated into a 96-well cell culture plate, with three sub-wells in each group. A 96-well cell culture plate was prepared in advance for the drug susceptibility test in a concentration gradient arrangement. Serum-free medium containing small molecules was added according to a concentration gradient (50, 25, 12.5, 6.25, 3.125, 1.56, 0.78, and 0 µM) and the culture was continued for 48 h. Thereafter, CCK-8 reagent was added and cells were incubated for 2 h at 37 °C, and the absorbance value at 450 nm wavelength was detected using a microplate reader. The tumor cell proliferation inhibition curve was drawn and the EC_50_ value of the small-molecule compound was calculated.

### Immunofluorescence assays

Immunofluorescence was used to detect the cellular localization of KK-LC-1. Briefly, cells were grown on glass coverslips, fixed in 4% paraformaldehyde for 30 min at room temperature, then blocking in 5% BSA for 90 min at room temperature and incubated with respective KK-LC-1 antibodies overnight at 4 ℃. Cells were washed in PBS three times, and were stained with 4',6-diamidino-2-phenylindole (DAPI) (blue). Cells were visualized using an immunofluorescence microscope (Nikon Oplenic Lumicite 9000).

### Statistical analysis

Statistical analyses were completed by SPSS 25.0 software and GraphPad Prism 8.0.2. The comparison between measurement data groups was conducted using independent sample *t* tests. The relationships between KK-LC-1 expression and clinicopathological variables were analyzed by chi-square tests. Correlation analysis of KK-LC-1 with MAL2, MUC1, and CLDN2 expression was conducted using Pearson tests. Survival analysis of breast cancer patients was performed using the Kaplan–Meier method and COX regression analyses were used to explore independent prognostic predictors for DFS and OS. *P* values < 0.05 were considered to be statistically significant.

## Results

### Screening CTA proteins that were highly expressed in breast cancer tissues compared to normal tissues using TCGA data

Using TCGA database, we first analyzed the expression of 276 CTA proteins in breast cancer and normal tissues. We found that expression levels of 47 CTA proteins were significantly higher in breast cancer tissues than in normal breast tissues (screening criteria: LogFC > 2 and *P* < 0.05). These results are presented in Additional file 2.

### Screening the CTA proteins that were highly expressed in triple-negative breast cancer tissues compared to normal tissues using TCGA data

**Fig. 1:**
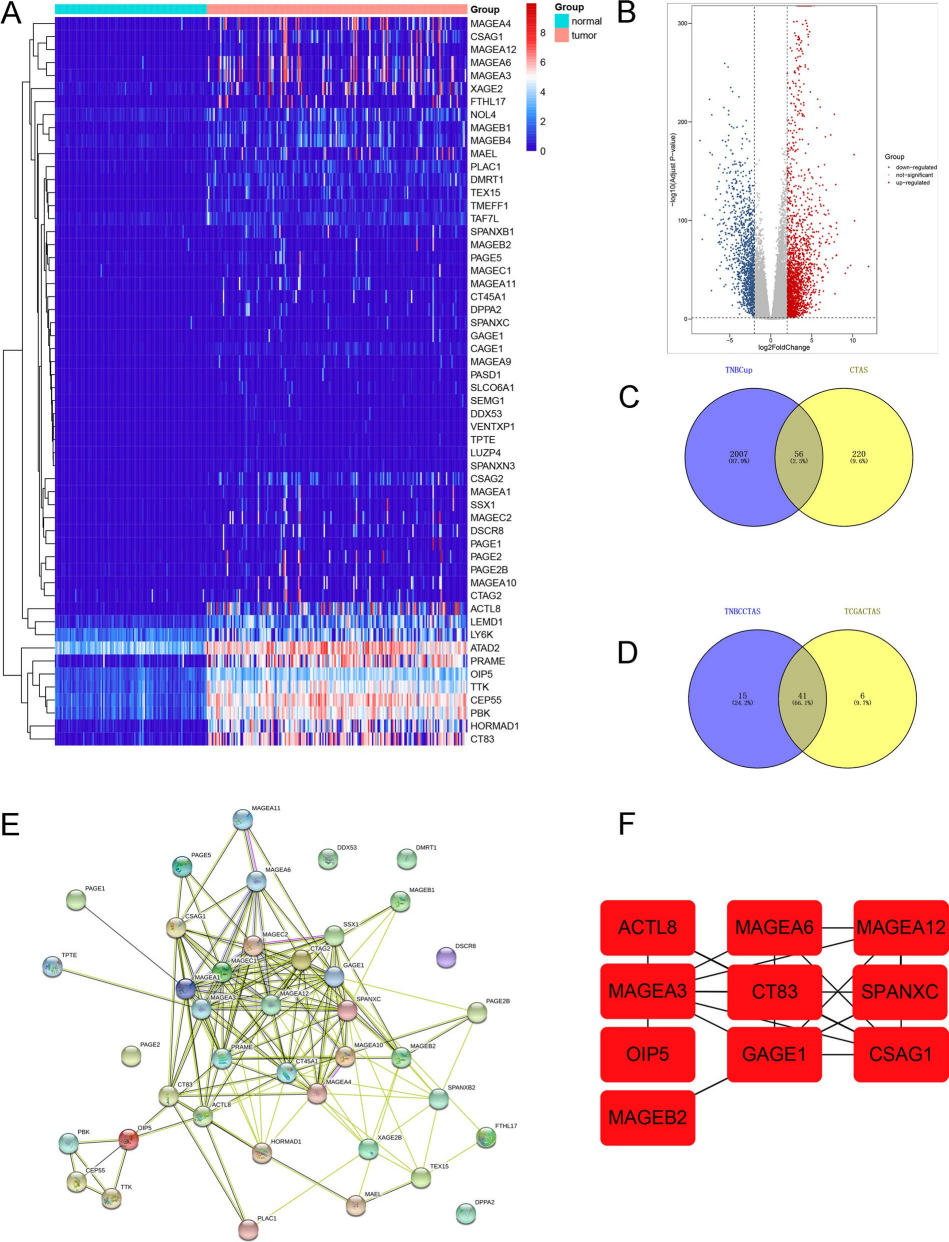
Expression of CTAs in triple-negative breast cancer data from TCGA database. **A** Heatmap of 56 CTAs in triple-negative breast cancer data from TCGA database. **B** Volcano plot of differentially expressed genes in triple-negative breast cancer and adjacent cancer data from TCGA database. **C** Venn diagram of the intersection of 276 CTAs and upregulated genes in triple-negative breast cancer data from TCGA database. **D** Venn diagrams were used to screen for CTAs with higher expression in overall breast cancer than in adjacent tumors and with higher expression in triple-negative breast cancer than in adjacent tumors. **E** Protein–protein interaction network diagram of 41 CTAs. F: Screening of hub genes among 41 CTAs

Since expression levels of CTAs were somewhat specific for triple-negative breast cancer subtypes, we further analyzed CTA expression in these tissues using TCGA data. We found the expression of 56 CTA proteins was significantly higher in triple-negative breast cancer tissues than in normal tissues (Fig. 1A–C). Further intersecting with the 47 CTAs in the above results, it was found that a total of 41 CTAs displayed higher expression in breast cancer than in adjacent cancer, and in triple-negative breast cancer than in adjacent cancer (Fig. 1D). For these 41 CTAs, we constructed a PPI network (Fig. 1E) and screened hub genes (Fig. 1F). By analyzing these ten genes and combining the results with published literature, we identified *CT83* as the target gene for our study. The protein encoded by *CT83* was KK-LC-1. For accuracy and completeness of research, we also screened data from other molecular subtypes from TCGA database for CTAs with higher expression in breast cancer than in adjacent tumors and found no KK-LC-1 (Additional file 3).

### Exploration and validation of KK-LC-1 expression in breast cancer and normal tissues

**Fig. 2:**
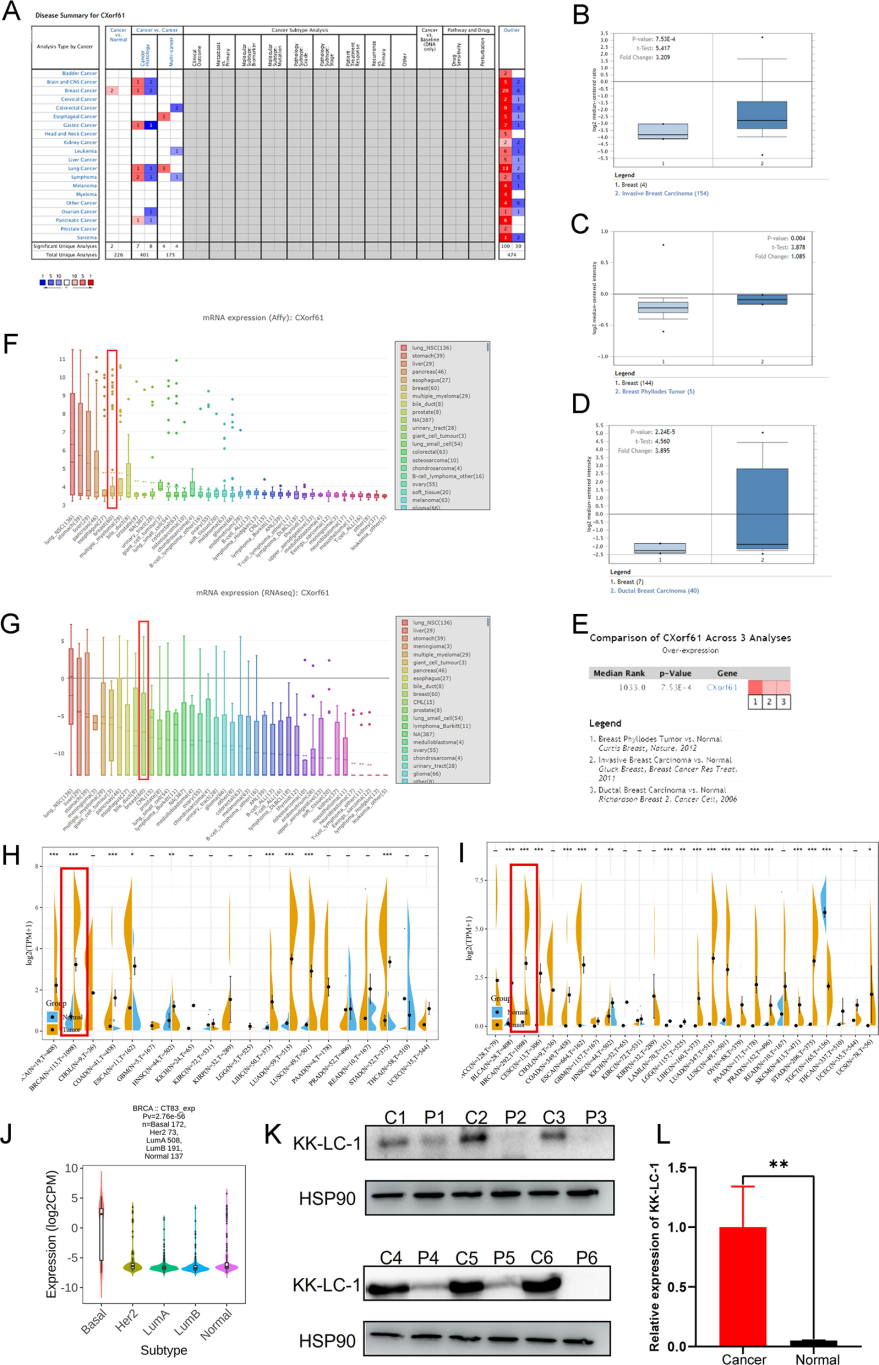
Exploration and validation of KK-LC-1 expression. **A** The Oncomine database was used to analyze Cxorf61 expression in various tumor tissues and adjacent tissues. **B** Cxorf61 expression was higher in invasive ductal carcinomas. **C** Cxorf61 expression was higher in phyllodes tumors. **D** Cxorf61 expression was higher in ductal breast cancer. **E** Integrative analysis of three studies B, C, and D showed that the expression of Cxorf61 in breast cancer tissues was higher. **F**, **G** CCLE database data indicated that Cxorf61 was highly expressed in breast cell lines using both methods (Affy and RNAseq). **H** The Sangerbox website explores *KK-LC-1* mRNA expression in breast cancer and adjacent normal tissues. **I** The Sangerbox website explores *KK-LC-1* mRNA expression in breast cancer and adjacent normal tissues by further integrating data from normal breast tissues in the GTEx database. **J** The TISIDB database shows *KK-LC-1* mRNA expression in different breast cancer molecular subtypes. **K**, **L** The expression of KK-LC-1 protein in six pairs of fresh triple-negative breast cancer and adjacent tissues was detected using western blotting

We first explored KK-LC-1 expression using bioinformatic tools. Oncomine data results suggested that the KK-LC-1 expression in breast cancer was higher than that in adjacent normal tissues (Fig. 2A). Using the results of three different research groups, it was found that in invasive ductal carcinoma, phyllodes tumor, and ductal breast cancer, the KK-LC-1 expression was higher than that in adjacent normal tumors (Fig. 2B–D). We performed an Oncomine meta-analysis of these three studies and obtained consistent results (Fig. 2E). The results of the CCLE database also showed that KK-LC-1 was highly expressed in breast cancer cell lines and was at the top of the analysis using both methods (Fig. 2F, G). We further explored KK-LC-1 expression using the Sangerbox tool. We also observed significantly higher expression in breast cancer tissues than in normal breast tissues (Fig. 2H). After further integrating the data of normal breast tissues in the GTEx database, it was found that the difference was more significant (Fig. 2I). Finally, we explored *KK-LC-1* mRNA expression in different breast cancer molecular subtypes using the TISIDB database. *KK-LC-1* mRNA expression was higher in triple-negative breast cancer than in other subtypes (Fig. 2J). The above results indicated that, using bioinformatics, we can preliminarily confirm that the KK-LC-1 expression in breast cancer was higher than that in adjacent normal tissues. We further collected six pairs of fresh triple-negative breast specimens and adjacent normal specimens. Table 1 presents the basic clinicopathological characteristics of the six patients. Western blotting revealed that KK-LC-1 protein expression was significantly higher in triple-negative breast cancer tissues than in adjacent normal tissues (Fig. 2K, L).

**Table 1:** Clinicopathological characteristics of six triple-negative breast cancer patients

	Patient 1	Patient 2	Patient 3	Patient 4	Patient 5	Patient 6
Gender	Female	Female	Female	Female	Female	Female
Age	46	42	45	57	49	64
T grade	2	1	1	2	1	2
N grade	0	0	0	0	1	0
M grade	0	0	0	0	0	0
Menopausal status	Premenopausal	Premenopausal	Premenopausal	Postmenopausal	Premenopausal	Premenopausal
Molecular subtype	Triple-negative breast cancer	Triple-negative breast cancer	Triple-negative breast cancer	Triple-negative breast cancer	Triple-negative breast cancer	Triple-negative breast cancer

### Exploration and validation of the prognostic effect of KK-LC-1 expression on breast cancer patients

**Fig. 3:**
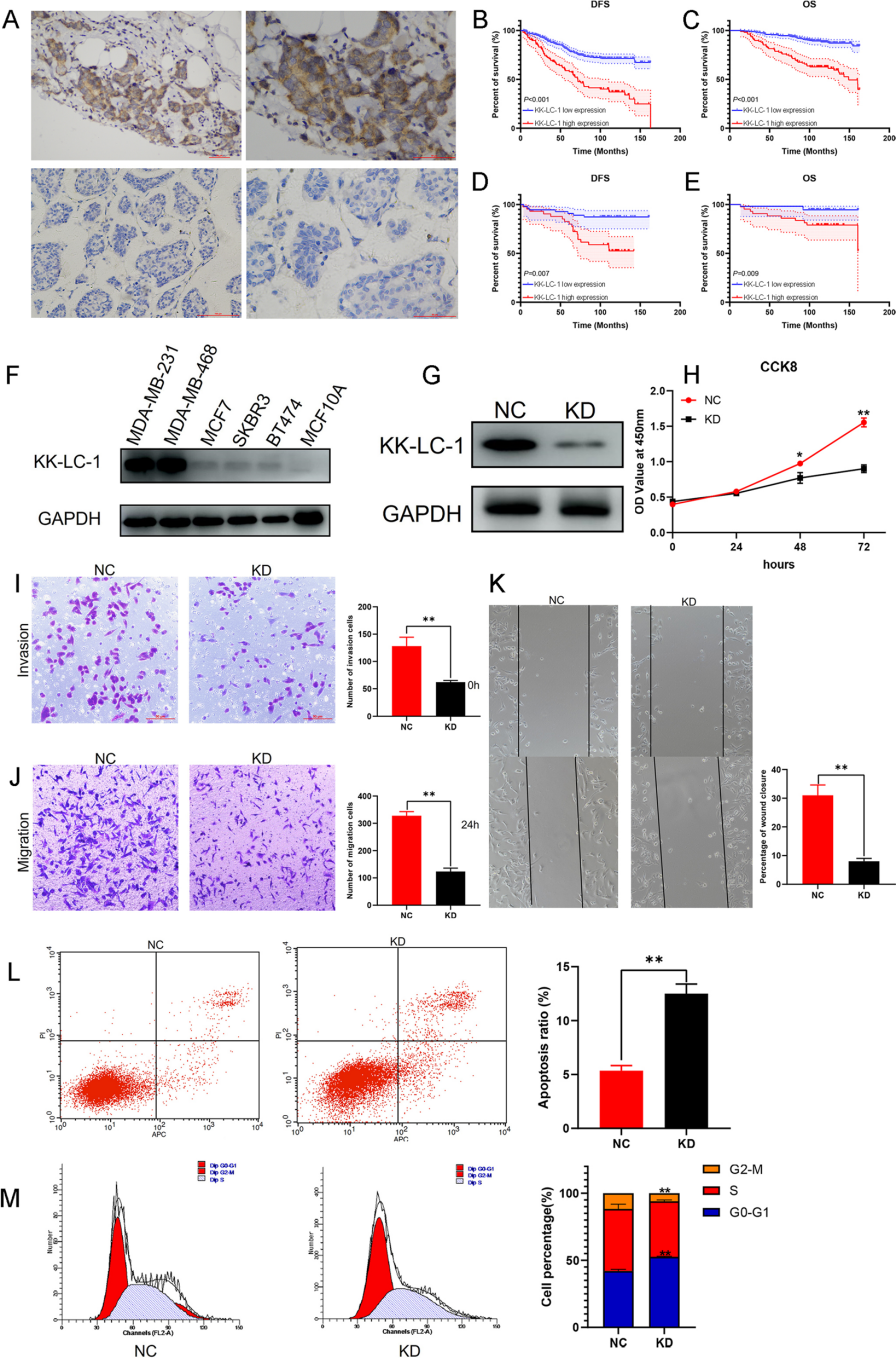
KK-LC-1 regulates the malignant biological behaviors of triple-negative breast cancer. **A** Representative images of high and low KK-LC-1 expression. (Left: ×200 magnification. Right: ×400 magnification). **B** Analysis of KK-LC-1 protein expression effects on DFS in 481 breast cancer patients. **C** Analysis of KK-LC-1 protein expression effects on the OS of 481 patients with breast cancer. **D** Analysis of KK-LC-1 expression effects on DFS in 98 triple-negative breast cancer patients. **E** Analysis of KK-LC-1 protein expression effects on OS in 98 triple-negative breast cancer patients. **F** The expression levels of KK-LC-1 in MDA-MB-231, MDA-MB-468, MCF7, SKBR3, BT474, and MCF10A cell lines were detected by western blotting. **G** The expression of KK-LC-1 in the MDA-MB-231 cell line was silenced by lentivirus and verified using western blotting. **H** Proliferation of MDA-MB-231/NC and MDA-MB-231/KD cells detected using the CCK8 assay. **I** Transwell assay to detect the invasive ability of MDA-MB-231/NC and MDA-MB-231/KD cells. **J** Transwell assay to detect the migration ability of MDA-MB-231/NC and MDA-MB-231/KD cells. **K** Differential analysis of the scratch healing ability of MDA-MB-231/NC and MDA-MB-231/KD cells. **L** Detection of apoptosis in MDA-MB-231/NC and MDA-MB-231/KD cells using flow cytometry. **M** Cell cycle detection in MDA-MB-231/NC and MDA-MB-231/KD cells using flow cytometry. (*P < 0.05, **P < 0.01)

Using the Kaplan–Meier plotter database, we found patients with high *KK-LC-1* mRNA expression had significantly shorter OS than patients with low *KK-LC-1* expression in two different studies (Additional file 4A, B). By analyzing other RNA-sequence data, we also found that patients with high *KK-LC-1* expression had a significantly shorter OS and post-progress survival (PPS) compared with patients with low *KK-LC-1* expression (Additional file 4C, D). To validate the prognostic effect of KK-LC-1 expression in breast cancer patients, we detected KK-LC-1 expression in 481 breast cancer specimens by immunohistochemistry. Typical pictures of high and low KK-LC-1 expression are shown in Fig. 3A. We analyzed the correlation between KK-LC-1 expression and clinicopathological characteristics (Table 2). We found high KK-LC-1 expression was significantly related to T grade, recurrence/distant metastasis, and death (*P* = 0.001, *P* = 0.001, and *P* = 0.001, respectively). However, it was not related to the patient age, N grade, or menopausal status. Furthermore, Kaplan–Meier analysis revealed that patients with high KK-LC-1 protein expression had significantly shorter DFS (*P* < 0.001; Fig. 3B) and OS (*P* < 0.001; Fig. 3C) than patients with low KK-LC-1 expression. We then explored the prognostic effect of KK-LC-1 expression in 98 triple-negative breast cancer patients. We also observed similar results (Fig. 3D, E). Cox regression analysis further validated our conclusion. Univariate and multivariate Cox regression analyses suggested that N grade, menopausal status, and KK-LC-1 expression were independent prognostic predictors for DFS, and N grade and KK-LC-1 expression were independent prognostic predictors for OS (Tables 3 and 4).

**Table 2:** The correlations between KK-LC-1 expression and clinicopathological characteristics

Variables	KK-LC-1 high expression (%)	KK-LC-1 low expression (%)	*P* value
No. of patients	108 (22.5)	373 (77.5)	–
Age			0.321
≤ 65	95 (88.0)	340 (91.2)	
> 65	13 (12.0)	33 (8.8)	
T grade			0.001
1	29 (26.9)	119 (31.9)	
2	63 (58.3)	237 (63.5)	
3	16 (14.8)	17 (4.6)	
N grade			0.071
0	51 (47.2)	227 (60.9)	
1	33 (30.6)	92 (24.7)	
2	8 (7.4)	16 (4.3)	
3	16 (14.8)	38 (10.1)	
Menopausal status			0.930
Postmenopausal	60 (55.6)	209 (56.0)	
Premenopausal	48 (44.4)	164 (44.0)	
Recurrence/distant metastasis			< 0.001
Yes	64 (59.3)	105 (28.2)	
No	44 (40.7)	268 (71.8)	
Death			< 0.001
Yes	48 (44.4)	41 (11.0)	
No	60 (55.6)	332 (89.0)

**Table 3:** Univariate and multivariate cox regression analyses of clinicopathological factors for DFS among these breast cancer patients

	Univariate analysis		Multivariate analysis	
	HR (95% CI)	*P* value	HR (95% CI)	*P* value
Age	1.398 (0.876–2.230)	0.160	NA	
T grade			NA	
1		0.450		
2	0.937 (0.674–1.302)	0.697		
3	1.328 (0.750–2.349)	0.330		
N grade			NA	
0		< 0.001		< 0.001
1	1.598 (1.095–2.331)	0.015	1.576 (1.080–2.300)	0.018
2	4.429 (2.565–7.647)	< 0.001	3.655 (2.110–6.331)	< 0.001
3	4.580 (3.079–6.812)	< 0.001	4.000 (2.681–5.970)	< 0.001
Menopausal status	1.460 (1.068–1.995)	0.018	1.438 (1.049–1.970)	0.024
KK-LC-1 expression	2.921 (2.136–3.993)	< 0.001	2.572 (1.875–3.528)	< 0.001

NA: Non-analysis

**Table 4:** Univariate and multivariate cox regression analyses of clinicopathological factors for OS among these breast cancer patients

	Univariate analysis		Multivariate analysis	
	HR (95% CI)	*P* value	HR (95% CI)	*P* value
Age	1.437 (0.764–2.704)	0.260	NA	
T grade			NA	
1		0.232		
2	1.498 (0.923–2.431)	0.102		
3	1.628 (0.698–3.798)	0.260		
N grade				
0		< 0.001		< 0.001
1	2.053 (1.234–3.415)	0.006	1.843 (1.106–3.070)	0.019
2	4.213 (1.936–9.170)	< 0.001	2.946 (1.345–6.453)	.007
3	4.101 (2.371–7.095)	< 0.001	3.350 (1.928–5.821)	< 0.001
Menopausal status	1.290 (0.844–1.972)	0.239	NA	
KK-LC-1 expression	4.435 (2.922–6.731)	< 0.001	3.844 (2.517–5.871)	< 0.001

NA: Non-analysis

### KK-LC-1 regulates the malignant biological behavior of triple-negative breast cancer cells

We found that the expression of KK-LC-1 was significantly higher in the triple-negative breast cancer cell lines MDA-MB-231 and MDA-MB-468 than in other cell lines (Fig. 3F). We silenced KK-LC-1 expression in MDA-MB-231 cells and validated this by western blotting (Fig. 3G). Using in vitro functional assays, we found that KK-LC-1 silencing significantly inhibited proliferation, invasion, migration, and scratch healing abilities of MDA-MB-231 cells (Fig. 3I–K). In addition, KK-LC-1 silencing significantly increased the ratio of cell apoptosis and arrested the cell cycle in the G0–G1 phase (Fig. 3L–M). The silencing of KK-LC-1 in MDA-MB-468 cells (Additional file 5A) also significantly inhibited breast cancer cell proliferation, invasion, migration, and scratch-healing abilities (Additional file 5B–H). In addition, KK-LC-1 silencing significantly increased the ratio of apoptotic cells and arrested the cell cycle in the S phase in MDA-MB-468 cells (Additional file 5I–L).

To deeply explore the effect of KK-LC-1 on the malignant biological behaviors of triple-negative breast cancer, we overexpressed KK-LC-1 in MDA-MB-231/KD cells to perform function rescue experiment. The overexpression efficiency of KK-LC-1 was shown in Additional file 6A. Then, we detected the effect of KK-LC-1 overexpression on cell proliferation, invasion, migration, scratch healing ability, apoptosis and cell cycle. As a result, we found that KK-LC-1 expression recovery can significantly increase the cell proliferation ability, invasion ability, migration ability, and scratch healing ability. And also, can significantly decrease the ratio of cell apoptosis and arrest the cell cycle in G0-G1 phase. These results were shown in Additional file 6B–G.

**Fig. 4:**
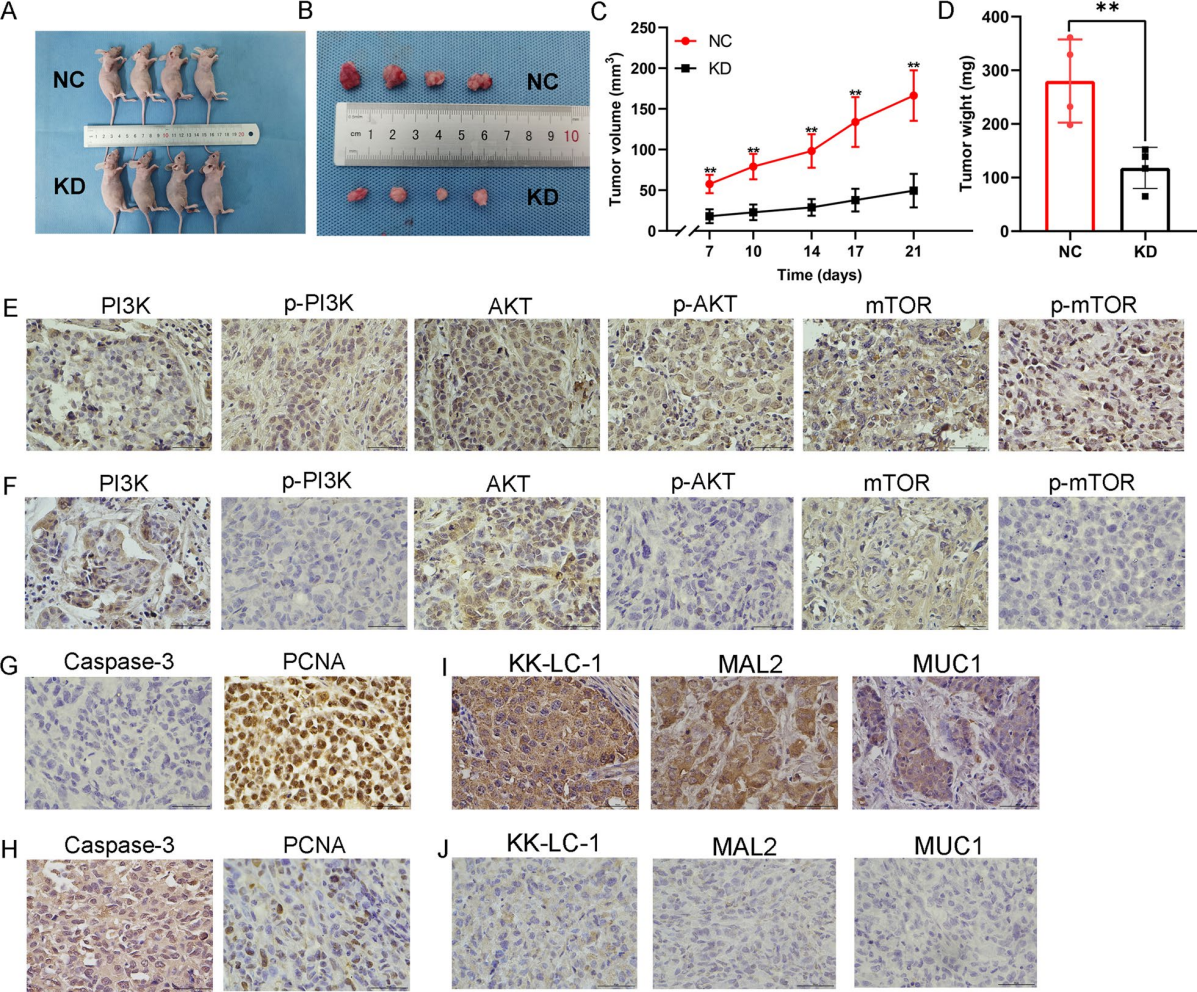
In vivo experiments presented that KK-LC-1 can regulate the malignant biological behaviors of triple-negative breast cancer. **A** In vivo tumorigenic models of NC and KD nude mice were successfully established. **B** Display of tumorigenic specimens in NC KD nude mice. **C** The volume change of tumor formation in nude mice. **D** Weight of tumor formation in nude mice. **E** Detection the expression of PI3K/AKT/mTOR pathway related proteins by immunohistochemistry in NC tumors. **F** Detection the expression of PI3K/AKT/mTOR pathway related proteins by immunohistochemistry in KD tumors. **G** Detection the expression of Caspase-3 and PCNA by immunohistochemistry in NC tumors. **H** Detection the expression of Caspase-3 and PCNA by immunohistochemistry in KD tumors. **I** Detection the expression of KK-LC-1, MAL2, MUC1 by immunohistochemistry in NC tumors. **J** Detection the expression of KK-LC-1, MAL2, MUC1 by immunohistochemistry in KD tumors. (**P < 0.01)

Thereafter, we injected MDA-MB-231/NC and MDA-MB-231/KD cells into nude mouse subcutaneous fat pads and found that KK-LC-1 silencing significantly reduced the volume and weight of tumors in nude mice by analyzing the changes in volume and mass after tumor formation in nude mice (Fig. 4A–D). These results confirmed that KK-LC-1 can regulate the biological characteristics of triple-negative breast cancer cells.

### KK-LC-1 silencing affects the expression of epithelial-mesenchymal transition (EMT) markers and breast cancer stem cells (BCSCs) markers

EMT and BCSCs play vital roles in the development of breast cancer [24, 25]. Therefore, we detected the expression of EMT and BCSCs markers in KK-LC-1 silenced MDA-MB-231 cells and normal control cells. We found that KK-LC-1 silencing significantly increased the expression of E-cadherin and decreased the expression of vimentin and Snail (Additional file 7A, B). These results suggest that EMT is involved in KK-LC-1 regulation of triple-negative breast cancer cells. We further detected the expression of BCSC markers in KK-LC-1-silenced MDA-MB-231 cells and normal control cells. We found that after KK-LC-1 silencing, the expression levels of Nanog, ALDH1A1, SOX2, OCT4, β-catenin, and pTAZ/TAZ were significantly decreased. However, the expression of nestin, and CD44 did not change (Additional file 7C–F). KK-LC-1 may regulate the stemness of triple-negative breast cancer cells, thereby exerting its regulatory effect on the biological characteristics of triple-negative breast cancer cells based on these results.

### Screening of key downstream genes of KK-LC-1 using NGS

We further explored the regulatory mechanism of KK-LC-1 in triple-negative breast cancer cell characteristics. We screened key downstream genes of KK-LC-1 using NGS. The heatmap and volcano map of NGS data are shown in Additional file 8A and B, respectively. After reviewing the literature, we selected and validated the mRNA expression of two oncogenes, *MAL2* and *MUC1* (Additional file 8C), as our downstream research targets of KK-LC-1, which were also tested in subsequent western blot experiments. We performed KK-LC-1 single-gene GSEA enrichment analysis using sequencing data. We found KK-LC-1 was enriched in the “Pathway in Cancer”, “Breast Cancer Pathway”, “Mammary Gland Epithelium Development”, “Wnt Beta-catenin Pathway”, “PI3K/AKT Signaling Pathway, “and “AKT, mTOR” (Additional file 8D–I).

### MAL2 expression, prognostic effect, and related pathway enrichment analysis in breast cancer

We first explored the expression of MAL2 using Sangerbox tools and found that MAL2 expression was higher in breast cancer tissues than in normal tissues (Additional file 9A). We integrated normal breast tissue data in the GTEx database and found that the difference was more significant (Additional file 9B). We also explored the expression of MAL2 using the GEPIA and UALCAN databases (Additional file 9C, D), and obtained similar results. Using the Kaplan–Meier plotter, we first performed survival analysis for MAL2. In two independent cohorts, patients with high *MAL2* mRNA expression had significantly shorter DFS and relapse-free survival (RFS) than patients with low *MAL2* expression (*P* = 0.01, Additional file 9E; *P* = 0.0062, Additional file 9F). In another RNA-sequence dataset, we observed that patients *MAL2* high expression was significantly related to shorter OS (*P* = 1.7e−05, Additional file 9G).

We obtained similar results using the UALCAN database. Patients with high *MAL2* mRNA expression had a significantly shorter lifetime than patients with low *MAL2* expression (*P* = 0.00018, Additional file 9H). Further subgroup analysis also showed that in different molecular subtype, menopausal status, and ethnic patient subgroups, high expression of *MAL2* was still significantly correlated with a shorter lifetime (Additional file 9I–K). Finally, we analyzed the results of the GEPIA and GSE9893 datasets and found that patients with high *MAL2* expression displayed worse survival outcomes (Additional file 9L, M). Using NGS data, we performed single-gene GSEA analysis of *MAL2* and found that it was enriched in the PI3K_AKT pathway (Additional file 9N).

### *MUC1* expression, prognostic effect, and related pathway enrichment analysis in breast cancer

We explored the expression of *MUC1* using Sangerbox tools and found that *MUC1* expression was higher in breast cancer tissues than in normal tissues (Additional file 10A). We integrated normal breast tissue data in the GTEx database and found that the difference was more significant (Additional file 10B). We also explored the expression of *MUC1* using the GEPIA and UALCAN databases (Additional file 10C, D), and obtained similar results. For the prognostic analysis of MUC1, we used data from the E-TABM-158, GSE1379, and GSE9398 datasets. We found that in the E-TABM-158 dataset, patients with high *MUC1* mRNA expression had significantly shorter OS, RFS, and disease-specific survival (DSS) than patients with low *MUC1* expression (*P* = 0.034, Additional file 10E; *P* = 0.044, Additional file 10F; *P* = 0.018, Additional file 10G). In the GSE1379 dataset, MUC1 high expression was significantly related to a shorter RFS (*P* = 0.032, Additional file 10H). In the GSE9398 dataset, MUC1 high expression was significantly related to a shorter OS (*P* < 0.001, Additional file 10I). Using NGS data, we performed single gene GSEA analysis of *MUC1* and found that it was also enriched in the PI3K_AKT pathway (Additional file 10J).

### KK-LC-1 regulates biological characteristics of triple-negative breast cancer by the MAL2/MUC1-C/PI3K/AKT/mTOR pathway

Based on the above results, we found that KK-LC-1 silencing can significantly decrease the expression of *MAL2* and *MUC1*, and data from KK-LC-1 silencing NGS suggested that it was enriched in the PI3K/AKT pathway. Interestingly, the GSEA results also predicted that *MAL2* and *MUC1* were all enriched in the PI3K/AKT pathway. The literature shows that MAL2 protein can interact with MUC1-C and positively regulate its expression in breast cancer [26]. Besides, it was also found that MAL2 can promote the proliferation, invasion, and metastasis of breast cancer cells via the EMT pathway [27]. The role of MUC1-C is more powerful and can be involved in several classical downstream pathways of breast cancer, including the PI3K/AKT/mTOR pathway [28]. Based on the literature and sequencing results of this study, we speculated that KK-LC-1 may regulate the biological characteristics of triple-negative breast cancer cells through the MAL2/MUC1-C/PI3K/AKT/mTOR pathway. To validate that, we firstly detected the expression of PI3K/AKT/mTOR pathway-related proteins in NC and KD tumors by immunohistochemistry assay. We found that both PI3K, AKT and mTOR were positively expressed in NC and KD tumors. However, p-PI3K, p-AKT and p-mTOR were only positively expressed in NC tumors, and were negatively expressed in KD tumors (Fig. 4E, F). Besides, we further detected the expression of proliferation/apoptotic markers in NC tumors and KD tumors by immunohistochemistry assay. Among the proliferation markers, we selected PCNA [29, 30]. Among the apoptotic markers, we selected Caspase-3 [31, 32]. In NC tumors, Caspase-3 was negatively expressed, and PCNA had strong positive expression. In KD tumors, Caspase-3 was positively expressed, and PCNA had week positive expression. These results were presented in Fig. 4G, H. It was also consistent with the results of in vitro functional experiments. Using these tumor samples, we also detected the expression of KK-LC-1, MAL2 and MUC1. In 4 pairs of NC tumors and KD tumors, we got similar results. The expression of KK-LC-1, MAL2 and MUC1 were positive in 4 NC tumors and were negative in 4 KD tumors. Therefore, there was a significantly positive correlations between KK-LC-1 protein expression and MAL2, MUC1 protein expression. The typical pictures of KK-LC-1, MAL2 and MUC1 positive and negative expression were shown in Fig. 4I, J.

**Fig. 5:**
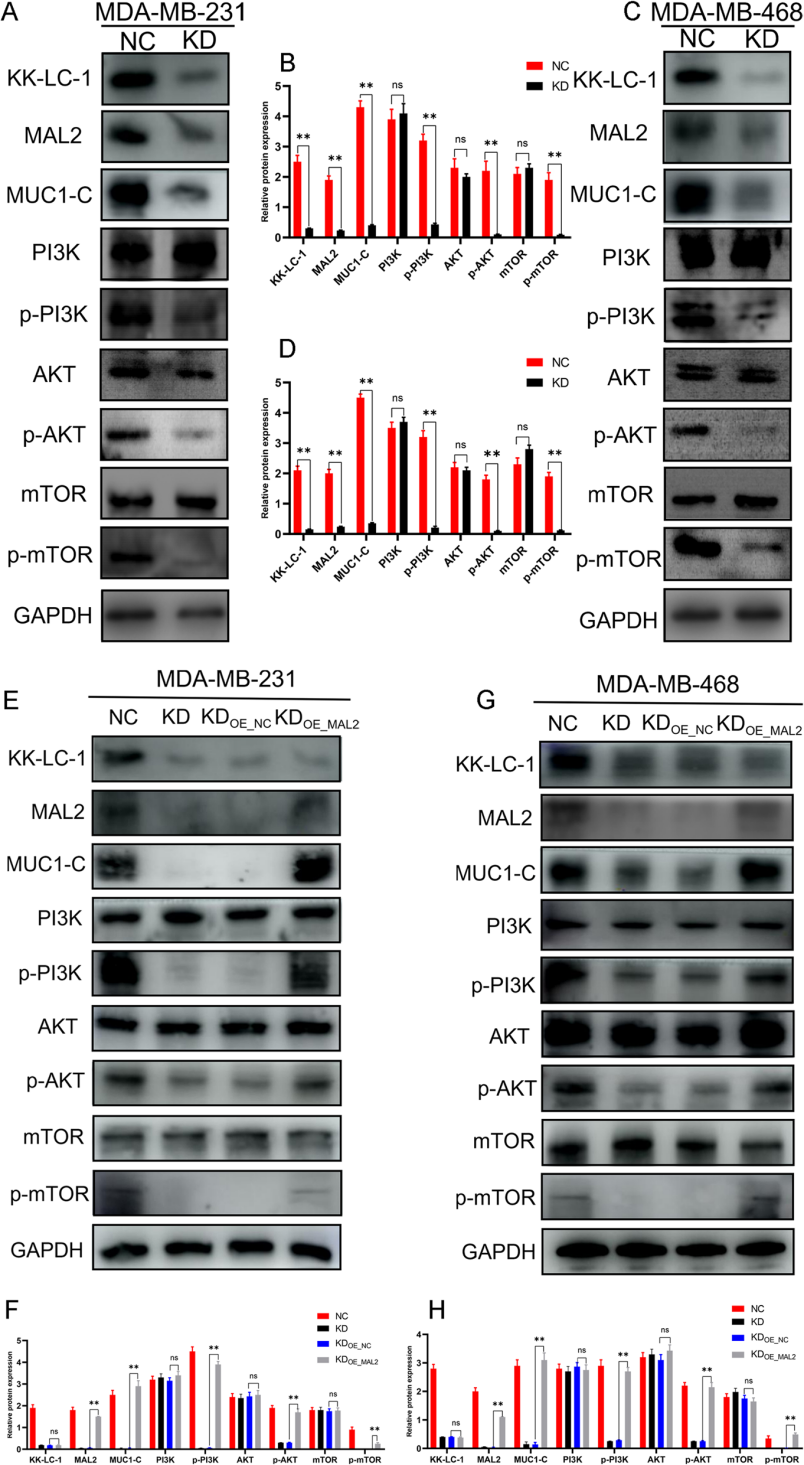
KK-LC-1 regulates the malignant biological behaviors of triple-negative breast cancer cells through the MAL2/MUC1-C/PI3K/AKT/mTOR pathway. **A**, **B** Detection of KK-LC-1, MAL2, MUC1-C, PI3K, p-PI3K, AKT, p-AKT, mTOR, and p-mTOR expression in MDA-MB-231/NC and MDA-MB-231/KD cells using western blotting (**P < 0.01). **C**, **D** Detection of KK-LC-1, MAL2, MUC1-C, PI3K, p-PI3K, AKT, p-AKT, mTOR, and p-mTOR expression in MDA-MB-468/NC and MDA-MB-468/KD cells using western blotting (**P < 0.01). **E**, **F** Detection of KK-LC-1, MAL2, MUC1-C, PI3K, p-PI3K, AKT, p-AKT, mTOR, and p-mTOR expression in MDA-MB-231/NC, MDA-MB-231/KD, MDA-MB-231/KD_OE_NC_, and MDA-MB-231/KD_OE_MAL2_ cells using western blotting (**P < 0.01). MDA-MB-231/KD_OE_NC_ cells: a group of empty plasmid-transfected KK-LC-1-silenced MDA-MB-231 cell lines. MDA-MB-231/KD_OE_MAL2_ cells: a group of MAL2 overexpression plasmid-transfected KK-LC-1-silenced MDA-MB-231 cell lines **G**, **H** Detection of KK-LC-1, MAL2, MUC1-C, PI3K, p-PI3K, AKT, p-AKT, mTOR, and p-mTOR expression in MDA-MB-468/NC, MDA-MB-468/KD, MDA-MB-468/KD_OE_NC_, and MDA-MB-468/KD_OE_MAL2_ cells using western blotting (**P < 0.01). MDA-MB-468/KD_OE_NC_ cells: a group of empty plasmid-transfected KK-LC-1-silenced MDA-MB-468 cell lines. MDA-MB-468/KD_OE_MAL2_ cells: a group of MAL2 overexpression plasmid-transfected KK-LC-1-silenced MDA-MB-468 cell lines

Additionally, we determined the expression of these proteins using western blotting in MDA-MB-231/KD, MDA-MB-468/KD, and normal control cells. We found that KK-LC-1 silencing significantly decreased the expression of MAL2, MUC1-C, p-PI3K/PI3K, p-AKT/AKT, and p-mTOR/mTOR (Fig. 5A–D). To further prove that MAL2/MUC1-C is an important downstream regulatory pathway of KK-LC-1, we overexpressed MAL2 in KK-LC-1-silenced cell lines and performed expression rescue experiments using MAL2 overexpression plasmid. We found MAL2 overexpression significantly increased MAL2, MUC1-C, p-PI3K/PI3K, p-AKT/AKT and p-mTOR/mTOR expression. However, KK-LC-1 expression did not change (Fig. 5E–H). We also performed rescue experiment to evaluate the effect of re-expressing KK-LC-1 in NC and KD cells on the mentioned pathways. This experiment included four groups: NC_OE_NC_ (The group of empty plasmid-transfected NC cell lines), NC_OE_KK-LC-1_ (The group of KK-LC-1 overexpression plasmid-transfected NC cell lines), KD_OE_NC_ (The group of empty plasmid-transfected KD cell lines), KD_OE_KK-LC-1_ (The group of KK-LC-1 overexpression plasmid-transfected KD cell lines). We detected the expression of KK-LC-1/MAL2/MUC1-C/PI3K/AKT/mTOR pathway-related proteins in these cells using western blotting. As a result, we found that compared with control group: NC_OE_NC_ and KD_OE_NC_, KK-LC-1 overexpression can significantly increase the expression of KK-LC-1, MAL2, MUC1-C, p-PI3K/PI3K, p-AKT/AKT, and p-mTOR/mTOR in MDA-MB-231 cells and MDA-MB-468 cells. These results were shown in Additional file 11.

**Fig. 6:**
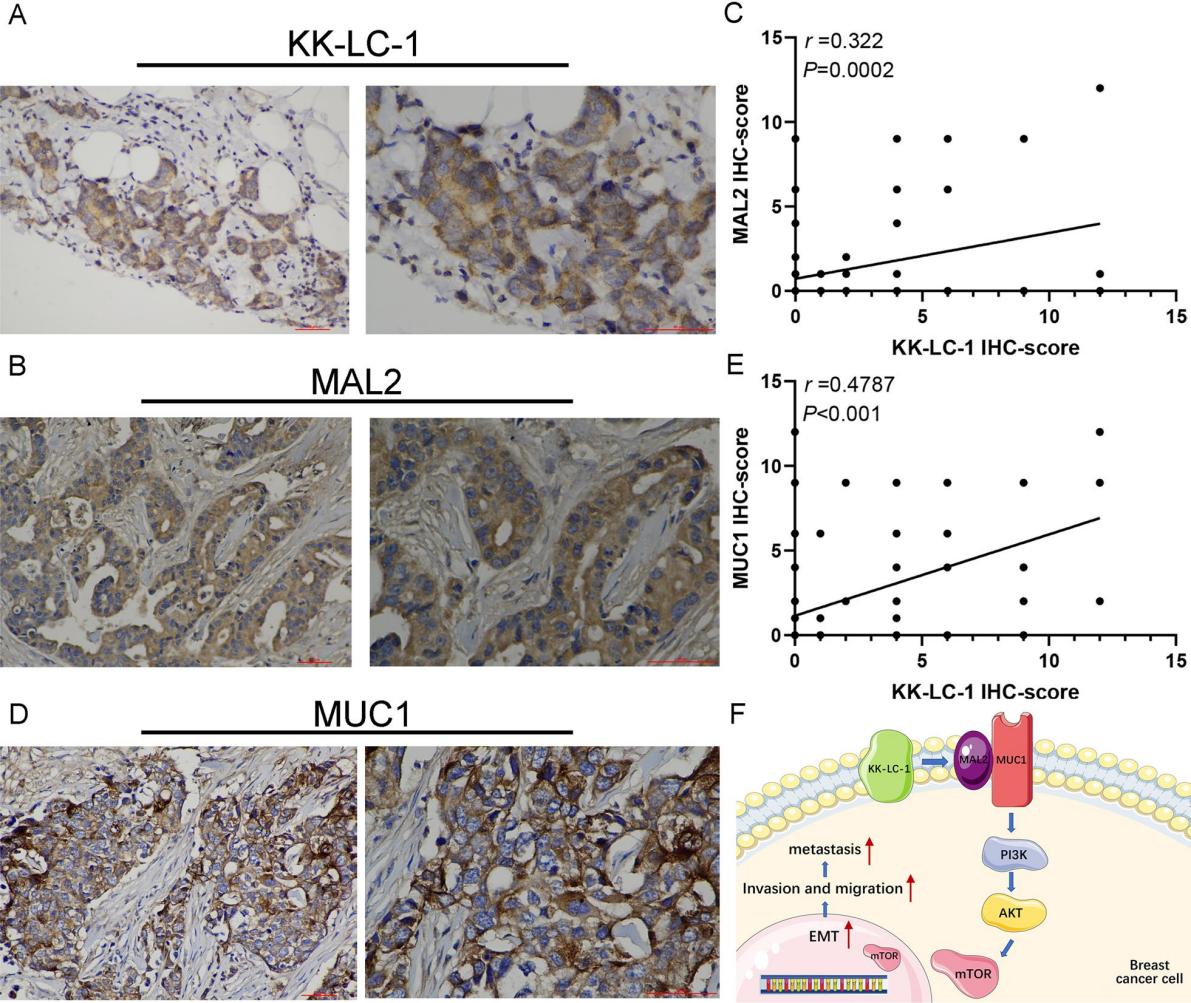
Validating the correlations between KK-LC-1 protein expression and MAL2 and MUC1 protein expression in triple-negative breast cancer specimens. **A** Representative pictures of high KK-LC-1 protein expression (Left: ×200 magnification. Right: ×400 magnification). **B** Representative images of high MAL2 protein expression (Left: ×200 magnification. Right: ×400 magnification). **C** Scatter plot of the correlation between KK-LC-1 and MAL2 protein expression. **D** Representative images of high MUC1 protein expression (Left: ×200 magnification. Right: ×400 magnification). **E** Scatter plot of the correlation between KK-LC-1 and MUC1 protein expression. **F** The schematic diagram demonstrates that KK-LC-1 regulates the biological behaviors of triple-negative breast cancer cells through the MAL2/MUC1-C/PI3K/AKT/mTOR pathway

To further validate the relationships between KK-LC-1, MAL2, and MUC1 expression, we detected the expression of KK-LC-1 (Fig. 6A), MAL2, and MUC1 in 126 triple-negative breast cancer clinical samples. KK-LC-1 expression was significantly positively correlated with MAL2 expression (Pearson test, r = 0.322; *P* = 0.0002; Fig. 6B, C). The expressions of KK-LC-1 and MUC1 were also significantly positively correlated (Pearson’s test, r = 0.4787; *P* < 0.0001; Fig. 6D, E). Based on these results, we can conclude that KK-LC-1 regulates the biological characteristics of triple-negative breast cancer cells through the MAL2/MUC1-C/PI3K/AKT/mTOR pathway. The schematic diagram demonstrates this pathway (Fig. 6F).

### KK-LC-1 may promote breast cancer liver metastasis by regulating the expression of CLDN2

When we analyzed NGS data, we found and validated that the mRNA level of the breast cancer liver metastasis gene *CLDN2* [33–35] was significantly decreased in KK-LC-1 silenced cells (Additional file 12A). We further validated the expression of CLDN2 using western blot in KK-LC-1-silenced MDA-MB-231 and MDA-MB-468 cells (Additional file 12B, C). Furthermore, we analyzed the correlation between KK-LC-1 and CLDN2 in triple-negative breast cancer specimens. KK-LC-1 expression was significantly positively related with CLDN2 expression (Pearson test, r = 0.3411; *P* < 0.0001; Additional file 12D, F). Therefore, we preliminarily concluded that KK-LC-1 may promote breast cancer liver metastasis by regulating the expression of CLDN2. However, we also want to explore the molecular mechanism that how KK-LC-1 can affect the expression of CLDN2.

After KK-LC-1 silencing, both the level of CLDN2 mRNA and protein were decreased. Therefore, we firstly speculated that KK-LC-1 can regulate the expression of CLDN2 at the level of transcription. Owing to KK-LC-1 was not a transcription factor, we conjectured that RNA-binding proteins (RBPs) may play an important role in the process. KK-LC-1 mRNA may competitively bind to certain RBP with CLDN2 mRNA to regulate the expression of CLDN2 protein. RBPs participated in almost all post-transcriptional gene regulation processes to determine the fate and functions of each transcript in cells. Aberrations in RBPs could alter RNA metabolism, transcriptome, and proteome of cells, which in turn, affected the growth, proliferative, and invasive capacities of cancer cells. RBPs can bind with mRNA to regulate the stabilization and degradation of mRNA [36–39].

Through ENCORI database (https://starbase.sysu.edu.cn/index.php), the candidate RBPs of KK-LC-1 mRNA and CLDN2 mRNA were sifted out as HNRNPL, PTBP1, SRSF1 and UPF1 (Additional file 12G). RBPs could mediate the stability of mRNA via binding to mRNA 3′UTR [40, 41]. RIP assay showed that only HNRNPL could bind to KK-LC-1 3′UTR among these four RIPs in MDA-MB-468 triple-negative breast cancer cells (Additional file 12H). Further, we validated that HNRNPL can also bind to CLDN2 3′UTR (Additional file 12I). Besides, RIP assay showed that KK-LC-1 silencing can significantly increase the binding ability of HNRNPL with CLDN2 3′UTR (Additional file 12J). Therefore, KK-LC-1 mRNA may competitively bind to HNRNPL with CLDN2 mRNA. Further experiments showed that KK-LC-1 silencing did not influence the expression of HNRNPL (Additional file 12K). However, HNRNPL silencing can significantly increase the expression of CLDN2 protein and mRNA (Additional file 12L, M). Furthermore, HNRNPL silencing notably increased the half-life of CLDN2 mRNA while that of β-actin was not affected (Additional file 12N). Collectively, we proved that HNRNPL could decrease the stability and expression of CLDN2 mRNA by bind to its 3′UTR. And, KK-LC-1 silencing can increase the binding between HNRNPL and CLDN2 mRNA, thus, the expression CLDN2 protein decreased. This may be the molecular mechanism that KK-LC-1 regulates CLDN2 expression.

### Screening of small-molecule compound targeting KK-LC-1 and drug sensitivity testing

**Fig. 7:**
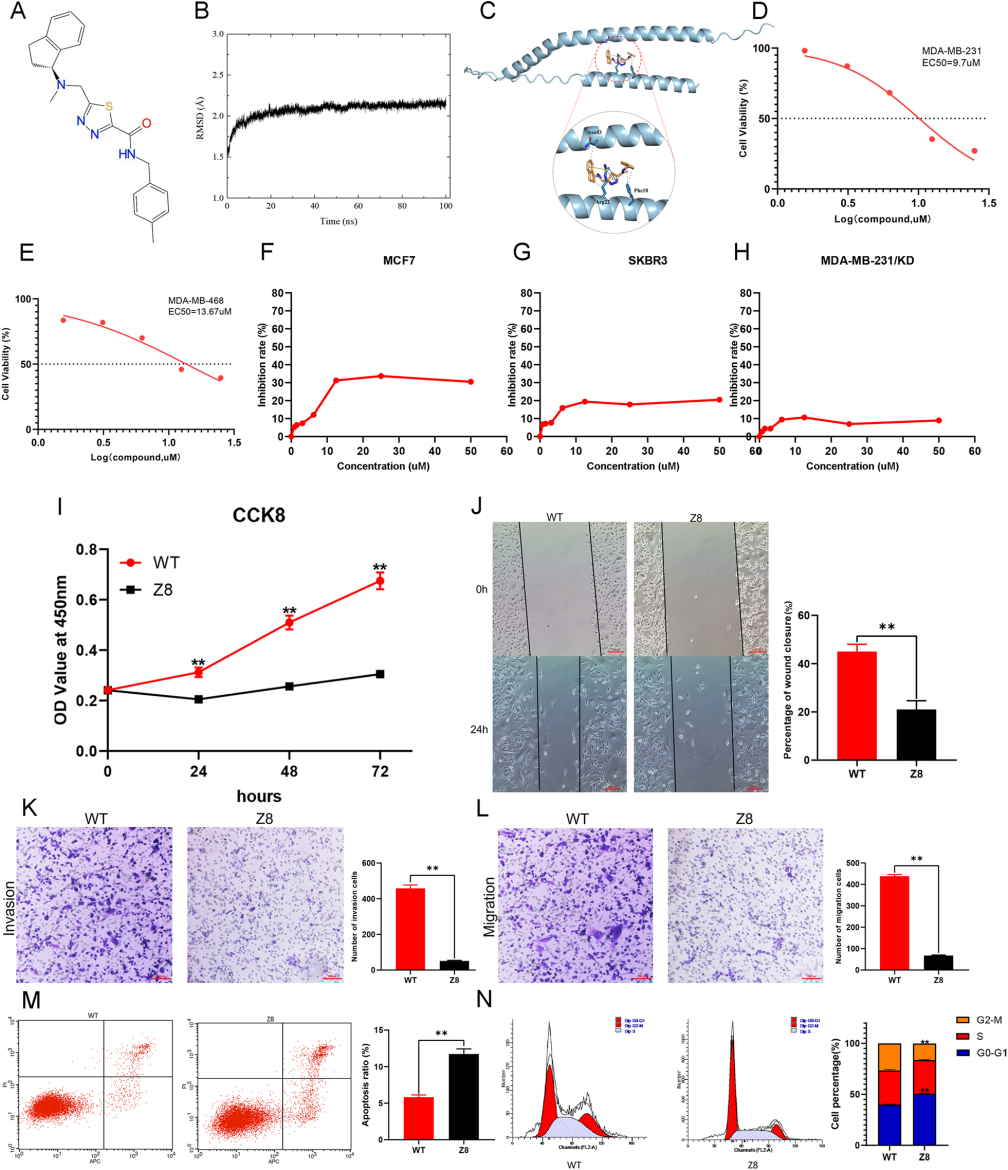
Drug susceptibility test of Z8 small-molecule compound in different breast cancer cell lines. And the inhibitory effect of Z8 on malignant biological behaviors of triple-negative breast cancer cells. **A** Z8 small-molecule compound molecular structure. **B**, **C** Molecular docking assay to analyze the binding ability of Z8 to KK-LC-1 pure protein. **D** Calculation of EC50 values of Z8 in MDA-MB-231 cells. **E** Calculation of EC50 values of Z8 in MDA-MB-468 cells. **F** Analysis of the inhibition rate of MCF7 cell proliferation by Z8 at different concentrations. **G** Analysis of the inhibition rate of SKBR3 cell proliferation by Z8 at different concentrations. **H** Analysis of the inhibition rate of MDA-MB-231/KD cell proliferation by Z8 at different concentrations. **I** Inhibitory effect of Z8 on the cell proliferation. **J** Inhibitory effect of Z8 on the wound healing ability. **K** Inhibitory effect of Z8 on the invasive ability. **L** Inhibitory effect of Z8 on the migration ability. **M** Z8 promotes apoptosis. **N** Z8 blocks cell cycle. (**P < 0.01)

By the AlphaFold Protein Structure database (www.alphafold.ebi.ac.uk), we obtained the three-dimensional structure of KK-LC-1 protein. Based on this structure, we screened the small-molecule compound Z839878730 (henceforth referred to as “Z8”) using the MCE compound library (Fig. 7A). Molecular docking results suggested a good binding ability between the KK-LC-1 protein and Z8 (Fig. 7B, C). We first tested the proliferation inhibition ability of Z8 on MDA-MB-231 and MDA-MB-468 cells using drug sensitivity testing. We found good proliferation inhibition ability of Z8 in triple-negative breast cancer cell lines. The EC_50_ value was 9.7 µM and 13.67 µM for MDA-MB-231 and MDA-MB-468 cells, respectively (Fig. 7D, E). Furthermore, we tested the proliferation inhibition ability of Z8 on low KK-LC-1 expression cells, such as MCF7, SKBR3, and MDA-MB-231/KD cells, and observed poor proliferation of Z8 in these cells. The highest concentrations of Z8 did not produce a 50% inhibition ratio (Fig. 7F–H). These results suggest that Z8 has good KK-LC-1 targeting and tumor-killing abilities.

### Z8 inhibits the malignant biological behaviors of triple-negative breast cancer cells

We performed in vitro functional experiments in MDA-MB-231 cells using the EC_50_ concentration of Z8 and observed that Z8 significantly inhibited the proliferation, scratch healing, invasion, and migration of MDA-MB-231 cells (Fig. 7I–L). Z8 also significantly increased the apoptosis ratio and arrested the cell cycle at the G0–G1 phase (Fig. 7M, N). Similar results were obtained for MDA-MB-468 cells using the EC_50_ concentration, indicating that Z8 can significantly inhibit the proliferation, scratch healing, invasion, and migration of MDA-MB-468 cells (Additional file 13A–G). Z8 also significantly increased the apoptosis ratio and arrested the cell cycle in the G0–G1 phase (Additional file 13H–K).

### Z8 has little tumor-killing effect on human normal mammary epithelial cells MCF10A

**Fig. 8:**
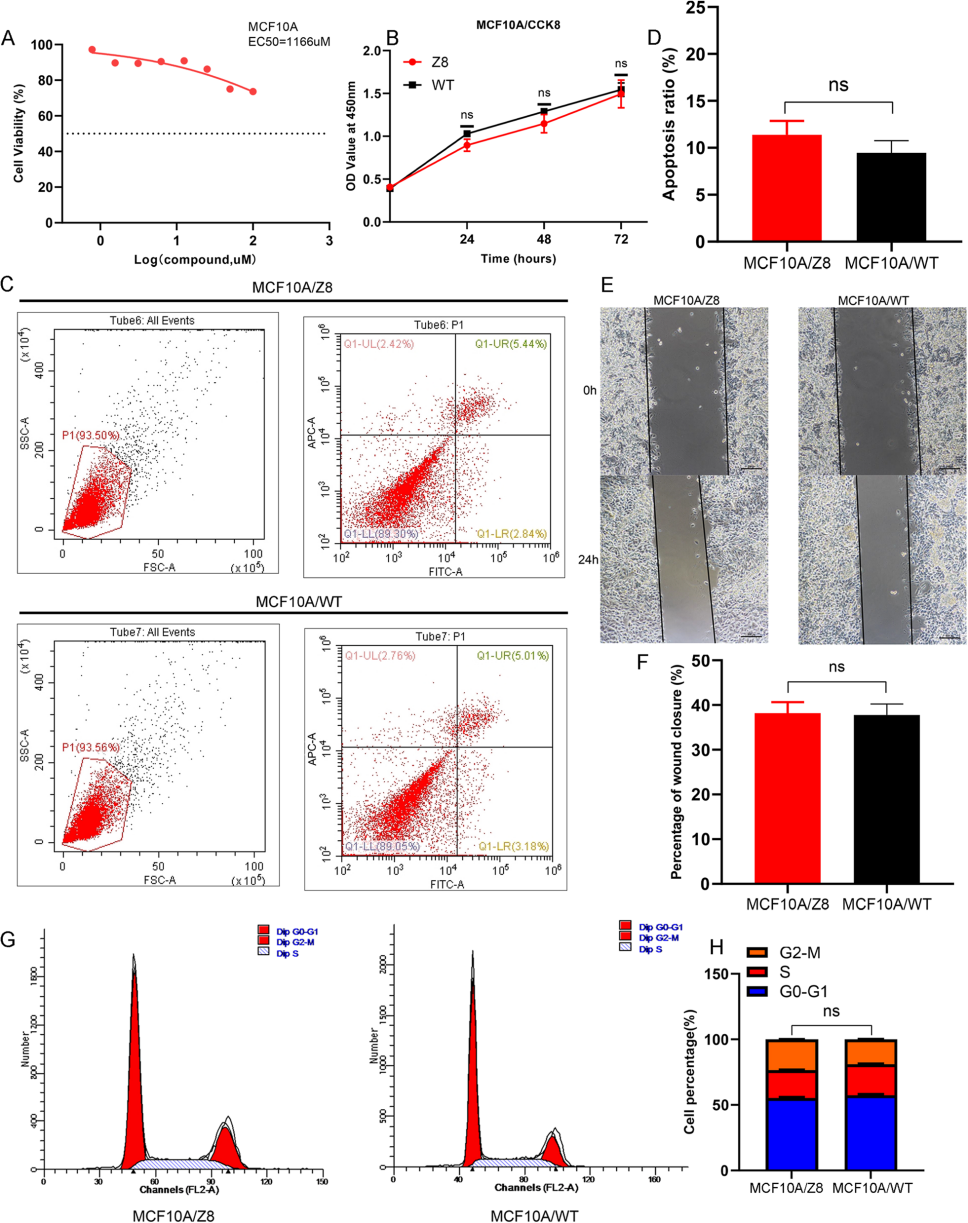
The tumor-killing effect of Z8 on human normal mammary epithelial cells MCF10A. **A** Calculation of EC50 values of Z8 in MCF10A cells. **B** The effect of Z8 on MCF10A cells proliferation. **C**, **D** The effect of Z8 on MCF10A cells apoptosis. **E**, **F** The effect of Z8 on wound healing ability of MCF10A cells. **G**, **H** The effect of Z8 on cell cycle of MCF10A cells

To further explore the tumor-killing effect of Z8 on untransformed breast cancer cells, we firstly performed drug sensitivity test of Z8 on MCF10A cells. However, in the group of the highest concentration of Z8 (100uM), the inhibition rate of cell proliferation was only nearly 20%. EC50 of Z839878730 on MCF10A cells by prism software was 1166uM (Fig. 8A). From this result, we can preliminarily come to a conclusion that Z8 possessed the effective ability of tumor-killing, however, had little tumor-killing effect on human normal mammary epithelial cells MCF10A. To further validate this point and the EC50 concentration of Z8 on triple-negative breast cancer cells had no cytotoxic effect on MCF10A cells, we treated MCF10A cells with Z8 to explore its effect on cell proliferation, apoptosis, scratch healing ability and cell cycle. The action concentration was according to the EC50 value of Z8 on triple-negative breast cancer cells (Among 9.7uM and 13.67uM, we selected 13.67uM as the action concentration.). As a result, compared with the control group, we found Z8 treatment cannot significantly affect MCF10A cell proliferation, apoptosis, scratch healing ability and cell cycle. These results were presented in Fig. 8B–H.

### Z8 can inhibit the malignant biological behaviors of triple-negative breast cancer cells by MAL2/MUC1-C/PI3K/AKT/mTOR pathway

**Fig. 9:**
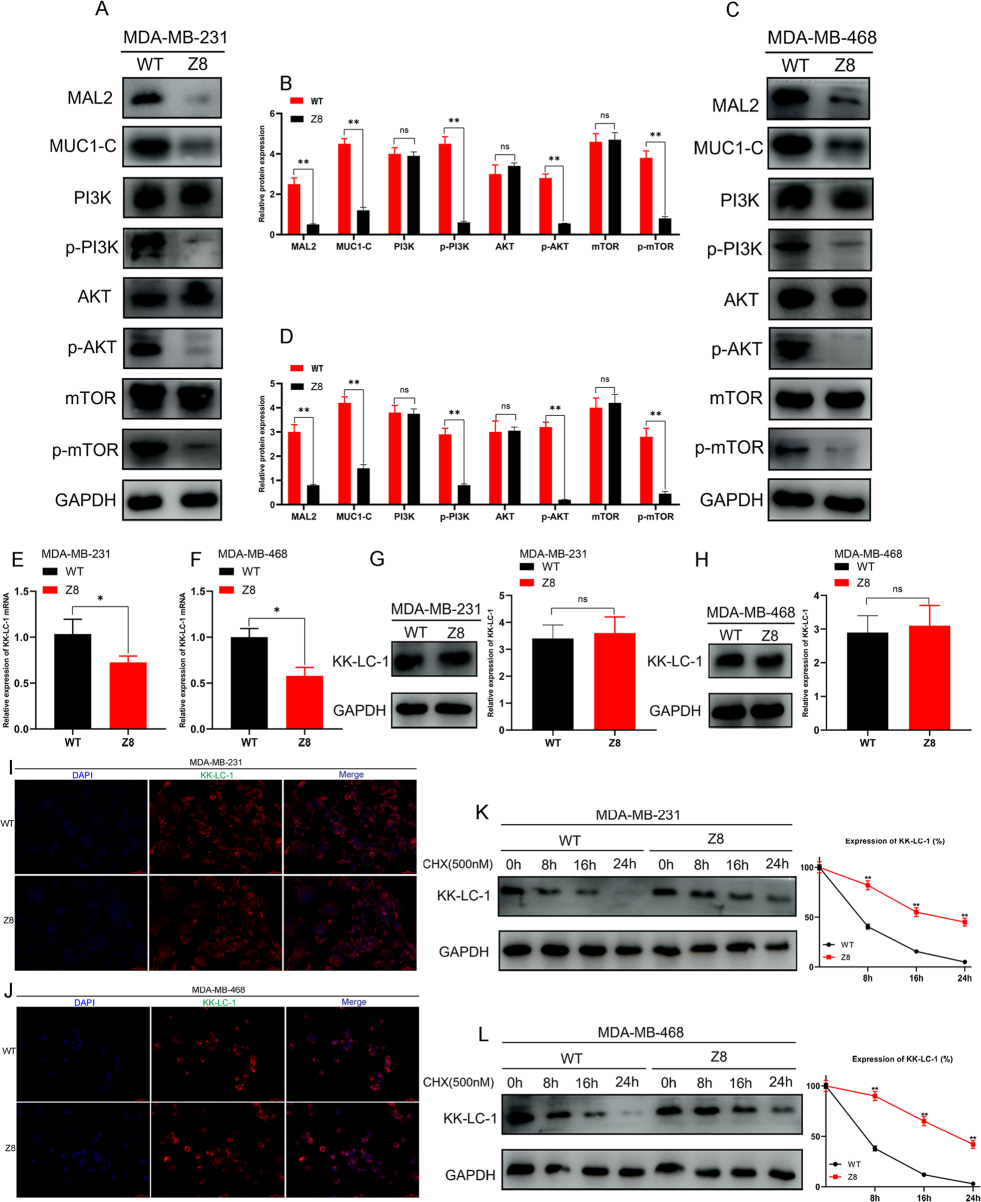
Z8 can inhibit the malignant biological behaviors of triple-negative breast cancer cells by MAL2/MUC1-C/PI3K/AKT/mTOR pathway. **A**, **B** Detection of MAL2, MUC1-C, PI3K, p-PI3K, AKT, p-AKT, mTOR, and p-mTOR expression in MDA-MB-231/WT and MDA-MB-231/Z8 cells using western blotting (**P < 0.01). MDA-MB-231/WT: DMSO treatment of MDA-MB-231 wild type cells. MDA-MB-231/Z8: Z8 treatment of MDA-MB-231 wild type cells. **C**, **D** Detection of MAL2, MUC1-C, PI3K, p-PI3K, AKT, p-AKT, mTOR, and p-mTOR expression in MDA-MB-468/WT and MDA-MB-468/Z8 cells using western blotting (**P < 0.01). MDA-MB-468/WT: DMSO treatment of MDA-MB-231 wild type cells. MDA-MB-468/Z8: Z8 treatment of MDA-MB-231 wild type cells. **E** Detection of KK-LC-1 mRNA expression in MDA-MB-231/WT and MDA-MB-231/Z8 cells using RT-qPCR (*P < 0.05). **F** Detection of KK-LC-1 mRNA expression in MDA-MB-468/WT and MDA-MB-468/Z8 cells using RT-qPCR (*P < 0.05). **G** Detection of KK-LC-1 expression in MDA-MB-231/WT and MDA-MB-231/Z8 cells using western blotting. **H** Detection of KK-LC-1 expression in MDA-MB-468/WT and MDA-MB-468/Z8 cells using western blotting. **I** Detection of KK-LC-1 intracellular localization in MDA-MB-231/WT and MDA-MB-231/Z8 cells using immunofluorescence assay. **J** Detection of KK-LC-1 intracellular localization in MDA-MB-468/WT and MDA-MB-468/Z8 cells using immunofluorescence assay. **K** Western blotting showing degradation of KK-LC-1 protein in MDA-MB-231/WT and MDA-MB-231/Z8 cells. **L** Western blotting showing degradation of KK-LC-1 protein in MDA-MB-468/WT and MDA-MB-468/Z8 cells

To further explore if Z8 can also influence the pathway of MAL2/MUC1-C/PI3K/AKT/mTOR, we treatment MDA-MB-231 and MDA-MB-468 triple-negative breast cancer cell lines with Z839878730. The concentration of Z8 on MDA-MB-231 and MDA-MB-468 was 9.7uM and 13.67uM (The EC50 value). As a result, compared with the control group, we found that Z8 can effectively decreased the expression of MAL2, MUC1-C, p-PI3K/PI3K, p-AKT/AKT, and p-mTOR/mTOR. This result was shown in Fig. 9A–D.

We also detected the effect of Z8 on KK-LC-1 levels and intracellular localization. In MDA-MB-231 and MDA-MB-468 cells, Z8 can significantly decrease the level of KK-LC-1 mRNA (Fig. 9E–F). However, Z8 cannot affect the expression of KK-LC-1 protein (Fig. 9G, H). As for the intracellular localization, by immunofluorescence assay, KK-LC-1 mainly located in cell membrane and partially located in cytoplasm. Z8 cannot affect the intracellular localization (Fig. 9I, J).

Owing to the expression of KK-LC-1 protein did not change, and the level of KK-LC-1 mRNA decreased after Z8 treatment, thus, we speculated that the binding between Z8 and KK-LC-1 may increase the half-time of KK-LC-1 protein, and shape negative feedback to reduce KK-LC-1 mRNA expression. To validate that, we conducted cycloheximide (CHX) protein degradation experiments. CHX was a kind of protein synthesis inhibitor. As a result, we found that Z8 treatment in MDA-MB-231 cells and MDA-MB-468 cells can significantly retard the degradation of KK-LC-1 protein compared with control group (Fig. 9K, L). This result may be the reason that Z8 did not affect the expression of KK-LC-1 protein, and decreased the level of KK-LC-1 mRNA.

## Discussion

Our results agree with those of Zhiqiang Chen regarding liver cancer [13]. The expression of KK-LC-1 was higher in breast cancer tissues than in normal breast tissues. High KK-LC-1 expression was significantly correlated with poor prognosis in breast cancer patients. Moreover, KK-LC-1 promoted the proliferation, invasion, migration, and scratch healing abilities of triple-negative breast cancer cells, and can also increase the apoptotic ratio and arrest the cell cycle. Silencing KK-LC-1 reduced tumor size and weight in nude mice. Therefore, we concluded that KK-LC-1 can regulate the malignant biological behavior of triple-negative breast cancer. Therefore, KK-LC-1 may be a potential therapeutic target for breast cancer.

EMT can enhance the migration and invasion of tumor cells, leading to tumor metastasis [42, 43]. Therefore, we analyzed changes in EMT marker expression after KK-LC-1 silencing and observed increased expression of E-cadherin [44] and decreased expression of vimentin [45] and snail [46]. Therefore, the EMT process is involved in KK-LC-1 regulation of invasion and metastasis of triple-negative breast cancer cells.

Many studies have shown that BCSCs are the initiating factors for breast cancer spread and metastasis [47, 48]. Conventional treatment methods can not only remove BCSCs but can also increase the BCSCs in the stem cell pool, thus indirectly leading to tumor recurrence [49]. Changes in the expression of various BCSCs markers are involved in these processes. We found that KK-LC-1 silencing decreased the expression of CSC markers Nanog [50], ALDH1A1 [51], SOX2 [52], OCT4 [53], and beta-catenin [54], and increased expression of pTAZ/TAZ [55]. This indicates that BCSCs are also involved in KK-LC-1 regulation of the invasion and metastasis of triple-negative breast cancer cells. However, the specific mechanism requires further study.

To analyze the regulatory mechanism of KK-LC-1 on the malignant biological behaviors of triple-negative breast cancer, we performed NGS at the mRNA level in MDA-MB-231/KD cells that stably silenced KK-LC-1 and MDA-MB-231/NC cells. By observing sequencing results and published literature, we preliminarily selected MAL2 and MUC1 as key downstream molecules of KK-LC-1. Studies have confirmed that MAL2 inhibits the presentation of tumor antigens by directly interacting with MHC-I complexes to promote the endocytosis of tumor antigens, thereby promoting the immune escape of breast cancer cells [56, 57]. Additionally, MAL2 has been found to be an independent prognostic predictor of breast cancer. MAL2 expression levels are inversely correlated with infiltration levels of lymphocytes in the breast cancer microenvironment [58]. MAL2 also promotes breast cancer cell proliferation, migration, and invasion by regulating the EMT [27]. We found that MAL2 expression was higher in breast cancer tissues than in normal tissues and was related to poor prognosis in breast cancer patients. We also found that MAL2 may be enriched in the PI3K/AKT pathway. Surprisingly, we found that MUC1 is the binding protein of MAL2 in breast cancer cells by reviewing the literature [26]. MAL2 was positively correlated with MUC1 expression, and MAL2 overexpression increases MUC1 expression. The C-terminal subunit of MUC1 (MUC1-C) is involved in multiple biological processes in breast cancer and is considered an oncoprotein. One study found that MUC1-C can promote the immune escape of triple-negative breast cancer cells by increasing the expression of PD-L1 [59]. In addition, MUC1-C has been found to be a excellent therapeutic target for breast cancer, and it participates in many classical pathways of cancer, including the MEK/ERK pathway [60], Wnt/β-catenin pathway [61], STAT/NF-κB pathway [62], and the PI3K/AKT pathway [28, 63–66]. MUC1-C can serve as a docking site for PI3K/AKT pathway activation. Overexpression of MUC1-C in DA3 murine breast cancer cells is associated with promotion of PI3K-dependent cell proliferation [67]. The MUC1-C cytoplasmic domain included a Y^20^HPM motif. When tyrosine is phosphorylated, this motif can serve as a binding site for the PI3K p85 SH2 domain. This indicates that the PI3K p85 subunit is tightly associated with the cytoplasmic domain of MUC1-C. In addition, researchers found that the mutation of the Tyr-20 amino acid in the cytoplasmic domain of MUC1-C blocked the binding of the PI3K p85 subunit to it. It can attenuate activation of the PI3K/AKT pathway induced by MUC1-C [68, 69]. Further studies also found that MUC1-C promotes glycolysis via the PI3K/AKT pathway [70]. The expression of MUC1-C was significantly positively associated with VEGF expression in clinical breast cancer samples [71]. Interestingly, the PI3K/AKT/mTOR pathway promotes *MUC1* gene transcription [28]. Since activation of the PI3K/AKT pathway can significantly promote proliferation and survival of breast cancer cells [64, 72–74], these results suggest that MUC1-C can promote the malignant progression of breast cancer by the PI3K/AKT pathway and may form a positive feedback loop.

We found that KK-LC-1 expression was significantly positively correlated with MAL2 and MUC1 expression in 126 triple-negative breast cancer specimens. Simultaneously, NGS data revealed that KK-LC-1 silencing could significantly reduce MAL2 and MUC1 expression, which was confirmed using RT-qPCR and western blotting. GSEA analysis of NGS data revealed that KK-LC-1 may be enriched in the PI3K/AKT pathway and MAL2 regulates MUC1-C expression, which regulates the PI3K/AKT pathway. Taken together, we speculated that KK-LC-1 may regulate the biological characteristics of triple-negative breast cancer via the MAL2/MUC1-C/PI3K/AKT pathway. Since mTOR is a canonical downstream molecule of PI3K/AKT, we also tested this hypothesis. We found that in MDA-MB-231 and MDA-MB-468 cells, KK-LC-1 silencing significantly decreased MAL2, MUC1-C, p-PI3K/PI3K, p-AKT/AKT, and p-mTOR/mTOR expression. To demonstrate that KK-LC-1 regulates the PI3K/AKT/mTOR pathway through the MAL2/MUC1-C pathway, we restored MAL2 expression in KK-LC-1-silenced cell lines. We found that MAL2 overexpression in MDA-MB-231/KD and MDA-MB-468/KD cells restored the expression of MUC-C, p-PI3K/PI3K, p-AKT/AKT, and p-mTOR/mTOR. However, KK-LC-1 expression did not change. Therefore, we conclude that KK-LC-1 regulates the biological characteristics of triple-negative breast cancer cells through the MAL2/MUC1-C/PI3K/AKT/mTOR pathway. This lays a theoretical foundation for the development of breast cancer drugs that target KK-LC-1.

Besides, KK-LC-1 was also significantly positively correlated with the expression of the breast cancer liver metastasis regulatory gene *CLDN2* and can regulate its expression by competitively binding to RBP-HNRNPL with CLDN2 mRNA. Kimbung et al*.* found that *CLDN2* expression was significantly higher in breast cancer liver metastasis foci than in other metastatic sites. CLDN2 promotes breast cancer metastasis. Compared to breast cancer patients without liver metastases, CLDN2 expression in the primary tumor of breast cancer patients with liver metastases was significantly increased. High expression of CLDN2 is closely associated with poor prognosis in breast cancer patients [35]. PM Siegel et al*.* found that high expression of CLDN2 is a sufficient and necessary condition for the colonization and growth of breast cancer cells in the liver. CLDN2 increases the ability of breast cancer cells with liver metastases to adhere to the extracellular matrix by upregulating integrin expression [33]. Tabariès et al*.* observed that CLDN2 can promote liver metastasis of colorectal cancer and that CLDN2 in colorectal cancer patient-derived extracellular vesicles can be used as a prognostic biomarker to predict the occurrence of surrogate liver metastases in colorectal cancer patients [75]. Therefore, KK-LC-1 may regulate breast cancer liver metastasis through CLDN2. However, further research is required to elucidate more specific mechanisms.

Finally, we screened the Z8 small-molecule compound targeting KK-LC-1. We observed good binding ability between Z8 and KK-LC-1 protein. Further in vitro experiments also found that Z8 has a low EC_50_ and can inhibit the malignant biological behaviors of KK-LC-1 high expressed triple-negative breast cancer cells MDA-MB-231 and MDA-MB-468 by MAL2/MUC1-C/PI3K/AKT/mTOR pathway. Moreover, this concentration had no cytotoxic effect on tumor cell lines and normal human mammary epithelial cell lines MCF10A that do not express KK-LC-1. The Z8 compound targets KK-LC-1 effectively and shows tumor cell killing ability. However, this study has some limitations. We did not use in vivo experiments to detect drug sensitivity of Z8 and conduct animal acute toxicity experiments to analyze the toxicity of Z8. Although we confirmed that the target of the Z8 compound is KK-LC-1, we have not further clarified the mechanism underlying the action between KK-LC-1 and Z8. Therefore, it is necessary to explore this topic in future research.

## Conclusion

In summary, we found that KK-LC-1 protein expression was higher in triple-negative breast cancer tissues than in normal tissues, and this high expression was significantly associated with poor survival outcomes in patients. KK-LC-1 promotes the malignant biological behavior of triple-negative breast cancer cells via the MAL2/MUC1-C/PI3K/AKT/mTOR pathway. We screened the Z8 small-molecule compound, which targets KK-LC-1 and has excellent tumor cell killing ability by inhibiting MAL2/MUC1-C/PI3K/AKT/mTOR pathway. We identified new therapeutic targets and provided novel treatment strategies for triple-negative breast cancer.

## Supplementary Information

**Additional file 1. **The antibody information used in this study.

**Additional file 2. **Expression of CTAs in TCGA breast cancer data. A: Heatmap of 47 CTAs in TCGA breast cancer data. B: Volcano plot of differentially expressed genes in breast cancer from TCGA database. C: Venn diagram of the intersection of 276 CTAs and upregulated genes in TCGA breast cancer data.

**Additional file 3. **Expression of CTAs in TCGA luminal A, luminal B, and HER2 + breast cancer data. A: Heatmap of the expression of 20 CTAs in TCGA luminal A type breast cancer data. B: Heatmap of the expression of 37 CTAs in TCGA luminal B type breast cancer data. C: Heatmap of the expression of 46 CTAs in TCGA HER2 positive breast cancer data.

**Additional file 4. **The role of KK-LC-1 expression in the prognosis of breast cancer patients using the Kaplan–Meier plotter database. A: The effect of increased *KK-LC-1* mRNA expression on OS was analyzed in a study including 1089 patients. B: The effect of increased *KK-LC-1* mRNA expression on OS was analyzed in a study including 2976 patients. C: The effect of increased *KK-LC-1* mRNA expression on OS was analyzed in a study that included 943 patients. D: The effect of increased *KK-LC-1* mRNA expression on PPS was analyzed in a study including 180 patients.

**Additional file 5. **KK-LC-1 regulates the malignant biological behaviors of MDA-MB-468 triple-negative breast cancer cells. A: The expression of KK-LC-1 in MDA-MB-468 cells was silenced by lentivirus and verified by western blotting. B: Proliferation of MDA-MB-468/NC and MDA-MB-468/KD cells determined by the CCK8 assay. C-D: Invasive ability of MDA-MB-468/NC and MDA-MB-468/KD cells determined by the Transwell assay. E–F: Migration ability of MDA-MB-468/NC and MDA-MB-468/KD cells determined by the Transwell assay. G, H: Differential analysis of the scratch healing ability of MDA-MB-468/NC and MDA-MB-468/KD cells. I, J: Detection of apoptosis in MDA-MB-468/NC and MDA-MB-468/KD cells using flow cytometry. K, L: Cell cycle detection of MDA-MB-468/NC and MDA-MB-468/KD cells using flow cytometry. (*P < 0.05, **P < 0.01)

**Additional file 6. **The effect of KK-LC-1 expression rescue on the malignant biological behaviors of MDA-MB-231 triple-negative breast cancer cells. A: Detection of KK-LC-1 expression in MDA-MB-231/NC, MDA-MB-231/KD, MDA-MB-231/KD_OE_NC_, and MDA-MB-231/KD_OE_KK-LC-1_ cells using western blotting. B: Proliferation of MDA-MB-231/NC, MDA-MB-231/KD, MDA-MB-231/KD_OE_NC_, and MDA-MB-231/KD_OE_KK-LC-1_ cells detected using the CCK8 assay. C: Transwell assay to detect the invasive ability of MDA-MB-231/NC, MDA-MB-231/KD, MDA-MB-231/KD_OE_NC_, and MDA-MB-231/KD_OE_KK-LC-1_ cells. D: Transwell assay to detect the migration ability of MDA-MB-231/NC, MDA-MB-231/KD, MDA-MB-231/KD_OE_NC_, and MDA-MB-231/KD_OE_KK-LC-1_ cells. E: Differential analysis of the scratch healing ability of MDA-MB-231/NC, MDA-MB-231/KD, MDA-MB-231/KD_OE_NC_, and MDA-MB-231/KD_OE_KK-LC-1_ cells. F: Detection of apoptosis in MDA-MB-231/NC, MDA-MB-231/KD, MDA-MB-231/KD_OE_NC_, and MDA-MB-231/KD_OE_KK-LC-1_ cells using flow cytometry. G: Cell cycle detection in MDA-MB-231/NC, MDA-MB-231/KD, MDA-MB-231/KD_OE_NC_, and MDA-MB-231/KD_OE_KK-LC-1_ cells using flow cytometry. MDA-MB-231/KD_OE_NC_ cells: a group of empty plasmid-transfected KK-LC-1-silenced MDA-MB-231 cell lines. MDA-MB-231/KD_OE_KK-LC-1_ cells: a group of KK-LC-1 overexpression plasmid-transfected KK-LC-1-silenced MDA-MB-231 cell lines.(**P < 0.01).

**Additional file 7. **Effects of KK-LC-1 silencing on the expression of EMT and BCSCs markers. A, B: Expression of E-cadherin, vimentin, and Snail in MDA-MB-231/NC and MDA-MB-231/KD cells was detected using western blotting (**P < 0.01). C, D: Expression of Nanog, ALDH1A1, SOX2, OCT4, and β-catenin in MDA-MB-231/NC and MDA-MB-231/KD cells was detected using western blot (**P < 0.01). E, F: The expression of TAZ, pTAZ, Nestin and CD44 in MDA-MB-231/NC and MDA-MB-231/KD cells was detected using western blotting (**P < 0.01).

**Additional file 8. **NGS results and GSEA analysis of MDA-MB-231/NC and MDA-MB-231/KD cells. A: Representative differential gene expression heatmap obtained using NGS. B: Volcano plot of differential gene expression using NGS. C: Validation the mRNA level of MAL2 and MUC1 in MDA-MB-231/NC and MDA-MB-231/KD cells by RT-qPCR. D-I: *KK-LC-1* single-gene GSEA enrichment analysis using NGS data.

**Additional file 9. **Bioinformatic methods integrated analysis of MAL2 expression and prognostic role in breast cancer, and GSEA analysis of *MAL2* gene. A: The Sangerbox tool was used to explore the expression of *MAL2* mRNA by analyzing the data from breast cancer and adjacent cancer tissues. B: The Sangerbox tool was used to explore the expression of *MAL2* mRNA by further integrating the data from normal breast tissues in the GTEx database. C: Analysis of *MAL2* mRNA expression in breast cancer and adjacent normal tissues using the GEPIA database. D: Analysis of *MAL2* mRNA expression in breast cancer and adjacent normal tissues using the UALCAN database. E: Effects of high *MAL2* mRNA expression on DFS in a study including 958 patients. F: The effect of high *MAL2* mRNA expression on RFS in a study including 2032 patients. G: Effects of high *MAL2* mRNA expression on OS in a study including 1089 patients. H: Effects of high *MAL2* mRNA expression on the survival of patients with breast cancer was analyzed using the UALCAN database. I: Effects of high *MAL2* mRNA expression on the survival of breast cancer patients with different molecular types, using the UALCAN database. J: Effects of high *MAL2* mRNA expression on the survival of breast cancer patients with different menopausal statuses, using the UALCAN database. K: Effects of high *MAL2* mRNA expression on the survival of breast cancer patients of different races, using the UALCAN database. L: Effects of high *MAL2* mRNA expression on OS in breast cancer patients using the GEPIA database. M: Effects of high *MAL2* mRNA expression on OS in patients with breast cancer in the GSE9893 database. N: GSEA analysis of *MAL2* gene.

**Additional file 10. **Bioinformatic methods integrated analysis of MUC1 expression and prognostic role in breast cancer, and GSEA analysis of *MUC1* gene. A: The Sangerbox tool was used to explore the expression of *MUC1* mRNA by analyzing the data from breast cancer and adjacent cancer tissues. B: The Sangerbox tool was used to explore the expression of *MUC1* mRNA by further integrating the data from normal breast tissues in the GTEx database. C: Analysis of *MUC1* mRNA expression in breast cancer and adjacent normal tissues using the GEPIA database. D: Analysis of *MUC1* mRNA expression in breast cancer and adjacent normal tissues using the UALCAN database. E: Effects of high *MUC1* mRNA expression on OS in the E-TABM-158 database. F: Effects of high *MUC1* mRNA expression on RFS in the E-TABM-158 database. G: Effects of high *MUC1* mRNA expression on DSS in the E-TABM-158 database. H: Effects of high *MUC1* mRNA expression on RFS in the GSE1379 database. I: Effects of high *MUC1* mRNA expression on OS in the GSE9398 database. J: GSEA analysis of *MUC1* gene.

**Additional file 11. **Rescue experiment to evaluate the effect of re-expressing KK-LC-1 in NC and KD cells on MAL2/MUC1-C/PI3K/AKT/mTOR pathway. A, B: Detection of KK-LC-1, MAL2, MUC1-C, PI3K, p-PI3K, AKT, p-AKT, mTOR, and p-mTOR expression in MDA-MB-231/NC_OE_NC_, MDA-MB-231/NC_OE_KK-LC-1_, MDA-MB-231/KD_OE_NC_ and MDA-MB-231/KD_OE_KK-LC-1_ cells using western blotting (**P < 0.01). C, D: Detection of KK-LC-1, MAL2, MUC1-C, PI3K, p-PI3K, AKT, p-AKT, mTOR, and p-mTOR expression in MDA-MB-468/NC_OE_NC_, MDA-MB-468/NC_OE_KK-LC-1_, MDA-MB-468/KD_OE_NC_ and MDA-MB-468/KD_OE_KK-LC-1_ cells using western blotting (**P < 0.01). NC_OE_NC_ (The group of empty plasmid-transfected NC cell lines). NC_OE_KK-LC-1_ (The group of KK-LC-1 overexpression plasmid-transfected KD cell lines). KD_OE_NC_ (The group of empty plasmid-transfected KD cell lines); KD_OE_KK-LC-1_ (The group of KK-LC-1 overexpression plasmid-transfected KD cell lines).

**Additional file 12: Fig. S15.** KK-LC-1 may promote breast cancer liver metastasis by regulating the expression of *CLDN2*. A: Detection of *CLDN2* mRNA expression in MDA-MB-231/NC, MDA-MB-231/KD MDA-MB-468/NC, MDA-MB-468/KD cells (*P < 0.05, **P < 0.01). B: Detection of CLDN2 protein expression in MDA-MB-231/NC and MDA-MB-231/KD cells using western blotting. C: Detection of CLDN2 protein expression in MDA-MB-468/NC and MDA-MB-468/KD cells using western blotting. D: Representative images of high KK-LC-1 protein expression (Left: 200× magnification. Right: 400× magnification). E: Representative images of high CLDN2 protein expression (Left: 200× magnification. Right: 400× magnification). F: Scatter plot of the correlation between KK-LC-1 and CLDN2 protein expression. G: The common RBPs of KK-LC-1 and CLDN2 were obtained through ENCORI. H: The binding between KK-LC-1 and candidate RBPs (HNRNPL, PTBP1, SRSF1 and UPF1) was assessed in MAD-MB-468 cells by RIP assay. (**P < 0.01). I: The binding between CLDN2 and HNRNPL was assessed in MAD-MB-468 cells by RIP assay. (**P < 0.01). J: The binding between CLDN2 and HNRNPL was analyzed by RIP assay with KK-LC-1 silencing. (**P < 0.01). K: Detection of KK-LC-1 and HNRNPL protein expression in MDA-MB-468/NC and MDA-MB-468/KD cells using western blotting. L: Detection of HNRNPL and CLDN2 protein expression in MDA-MB-468/sh_NC and MDA-MB-468/sh_HNRNPL cells using western blotting. M: Detection of CLDN2 mRNA expression in MDA-MB-468/sh_NC and MDA-MB-468/sh_HNRNPL cells using RT-qPCR. (**P < 0.01). N: The effect of HNRNPL on CLDN2 mRNA stability was assessed by RT-qPCR with the treatment of α-amanitin (an inhibitor of mRNA synthesis).

**Additional file 13. **Inhibitory effect of Z8 small-molecule compound on malignant biological behaviors of MDA-MB-468 cells. A: Inhibitory effect of Z8 on the proliferation. B, C: Inhibitory effect of Z8 on the wound healing ability. D, E: Inhibitory effect of Z8 on the invasive ability. F, G: Inhibitory effect of Z8 on the migration ability. H, I: Z8 promotes apoptosis. J, K: Z8 blocks the cell cycle. (**P < 0.01).

**Additional file 14. **The molecular mechanism that KK-LC-1 regulated the expression of MAL2. A: The common RBPs of KK-LC-1 and MAL2 were obtained through ENCORI. B: The binding between KK-LC-1 and candidate RBPs (EIF4A3, HNRNPL, PTBP1, SRSF1, SRSF3, U2AF2 and UPF1) was assessed in MAD-MB-468 cells by RIP assay. (**P < 0.01). C: The binding between MAL2 and EIF4A3, HNRNPL was assessed in MAD-MB-468 cells by RIP assay. (**P < 0.01). D: The binding between MAL2 and EIF4A3 was analyzed by RIP assay with KK-LC-1 silencing. (**P < 0.01). E: Detection of KK-LC-1 and EIF4A3 protein expression in MDA-MB-468/NC and MDA-MB-468/KD cells using western blotting. F: Detection of EIF4A3 and MAL2 protein expression in MDA-MB-468/sh_NC and MDA-MB-468/sh_EIF4A3 cells using western blotting. G: Detection of MAL2 mRNA expression in MDA-MB-468/sh_NC and MDA-MB-468/sh_EIF4A3 cells using RT-qPCR. (**P < 0.01). H: The effect of EIF4A3 on MAL2 mRNA stability was assessed by RT-qPCR with the treatment of α-amanitin (an inhibitor of mRNA synthesis).

**Additional file 15. **The molecular mechanism that KK-LC-1 directly regulated the expression of MUC1-C. A: Western blotting showing MAL2 protein levels in MA-MB-468/WT and MDA-MB-468/KO cells. MDA-MB-468/KO cells: MAL2 expression was knocked out by CRISPR-Cas9 in MDA-MB-468 cells. B: Western blotting showing MUC1-C and KK-LC-1 protein levels in MA-MB-468/WT and MDA-MB-468/KO cells. C: Western blotting showing KK-LC-1 and MUC1-C protein levels in KK-LC-1 silenced MDA-MB-468/KO cells. D: Western blotting showing MUC1-C protein levels in MDA-MB-468/KO cells treated with different combinations of cycloheximide, MG132 and bafilomycin at different time points. E: Western blotting showing MUC1-C protein levels in KK-LC-1 silenced MDA-MB-468/KO cells treated with MG132 and bafilomycin. F: Western blotting showing degradation of MUC1-C protein in KK-LC-1 overexpressed MDA-MB-468/KO cells and control MDA-MB-468/KO cells. G: Western blotting showing the expression of KK-LC-1, MAL2 and MUC1-C protein in KK-LC-1 overexpressed MDA-MB-468/KO cells and control MDA-MB-468/KO cells with the treatment of CHX. And immunoprecipitation assay showed that KK-LC-1 overexpression can promote the interaction between MAL2 and MUC1-C. OE_NC (The group of empty plasmid-transfected MDA-MB-468/KO cells). OE_KK-LC-1 (The group of KK-LC-1 overexpression plasmid-transfected MDA-MB-468/KO cells)

## Data Availability

These data and materials can be available from the corresponding author on reasonable request.

## Abbreviations

CTAs: Cancer/testis antigens
KK-LC-1: Kita-Kyushu lung cancer antigen-1
DFS: Disease-free survival
OS: Overall survival
TCGA: The Cancer Genome Atlas
PPI: Protein–protein interaction
GSEA: Gene set enrichment analysis
NGS: Next-generation sequencing
GEO: Gene Expression Omnibus
NC: Negative Control
RIP: RNA binding protein immunoprecipitation
DMSO: Dimethyl sulfoxide
DAPI: 4′,6-Diamidino-2-phenylindole
PPS: Post-progress survival
EMT: Epithelial-mesenchymal transition
BCSCs: Breast cancer stem cells
RFS: Relapse-free survival
DSS: Disease-specific survival
RBPs: RNA-binding proteins
CHX: Cycloheximide
