# Supplementary material for: Targeting KK-LC-1 inhibits malignant biological behaviors of triple-negative breast cancer
Source: J Transl Med. 2023 Mar 9;21:184. doi: 10.1186/s12967-023-04030-9 (PMC9996895; doi:10.1186/s12967-023-04030-9)
Supplement: Supplementary file 1 — Additional file 1. The antibody information used in this study. [file 12967_2023_4030_MOESM1_ESM.pdf]

Additional file 1 The antibody information used in this study

Antibodies	Source	Identifier
GAPDH	Affinity	#AF7021
Cxorf61	Proteintech	25708-1-AP
MAL2	Bioss	bs-7175R
MUC1	ABclonal Technology	A19081
Claudin 2	Bioss	bsm-33414M
CLDN2	ABclonal Technology	A14085
ALDH1A1	Proteintech	15910-1-AP
CD44	Proteintech	15675-1-AP
NANOG	Proteintech	14295-1-AP
Nestin	Proteintech	19483-1-AP
OCT4	Proteintech	11263-1-AP
SOX2	Proteintech	11064-1-AP
Beta Tubulin	Proteintech	66240-1-Ig
E-Cadherin	Proteintech	20874-1-AP
Vimentin	Proteintech	10366-1-AP
Snail	ABclonal Technology	A5243
WWTR1	Immunoway	YN1701
TAZ (phospho-Ser89)	Immunoway	YP1526
Beta Catenin	Proteintech	66379-1-Ig
PI3K p85/p55	Affinity	#AF6242
Phospho-PI3K p85 (Tyr458)[Tyr467]/p55 (Tyr199)	Affinity	#AF3242
AKT	Proteintech	60203-2-Ig
Phospho-AKT (Ser473)	Proteintech	66444-1-Ig
mTOR	Proteintech	66888-1-Ig
Phospho-mTOR (Ser2448)	Proteintech	67778-1-Ig
Horseradish-conjugated goat anti-rabbit IgG (H+L) (affinity purified)	Zhong Shan Jin Qiao Biotechnology	ZB-2301
Horseradish-conjugated goat anti-mouse IgG (H+L) (affinity purified)	Zhong Shan Jin Qiao Biotechnology	ZB-2305
MUC1	Abcam	Ab45167
Caspase-3	Proteintech	66470-2-Ig
PCNA	HUABIO	ET1605-38
EIF4A3	Proteintech	17504-1-AP
HNRNPL	Proteintech	18354-1-AP
PTBP1	Proteintech	12582-1-AP
SRSF1	Proteintech	12929-2-AP
SRSF3	Abcam	ab198291
U2AF2	Proteintech	15624-1-AP
UPF1	Proteintech	23379-1-AP
Anti-KK-LC-1/CT83/PE Conjugated antibody	Bioss	Bs-16747R-PE
