# Supplementary figures and images for: Targeting KK-LC-1 inhibits malignant biological behaviors of triple-negative breast cancer
Source: J Transl Med. 2023 Mar 9;21:184. doi: 10.1186/s12967-023-04030-9 (PMC9996895; doi:10.1186/s12967-023-04030-9)

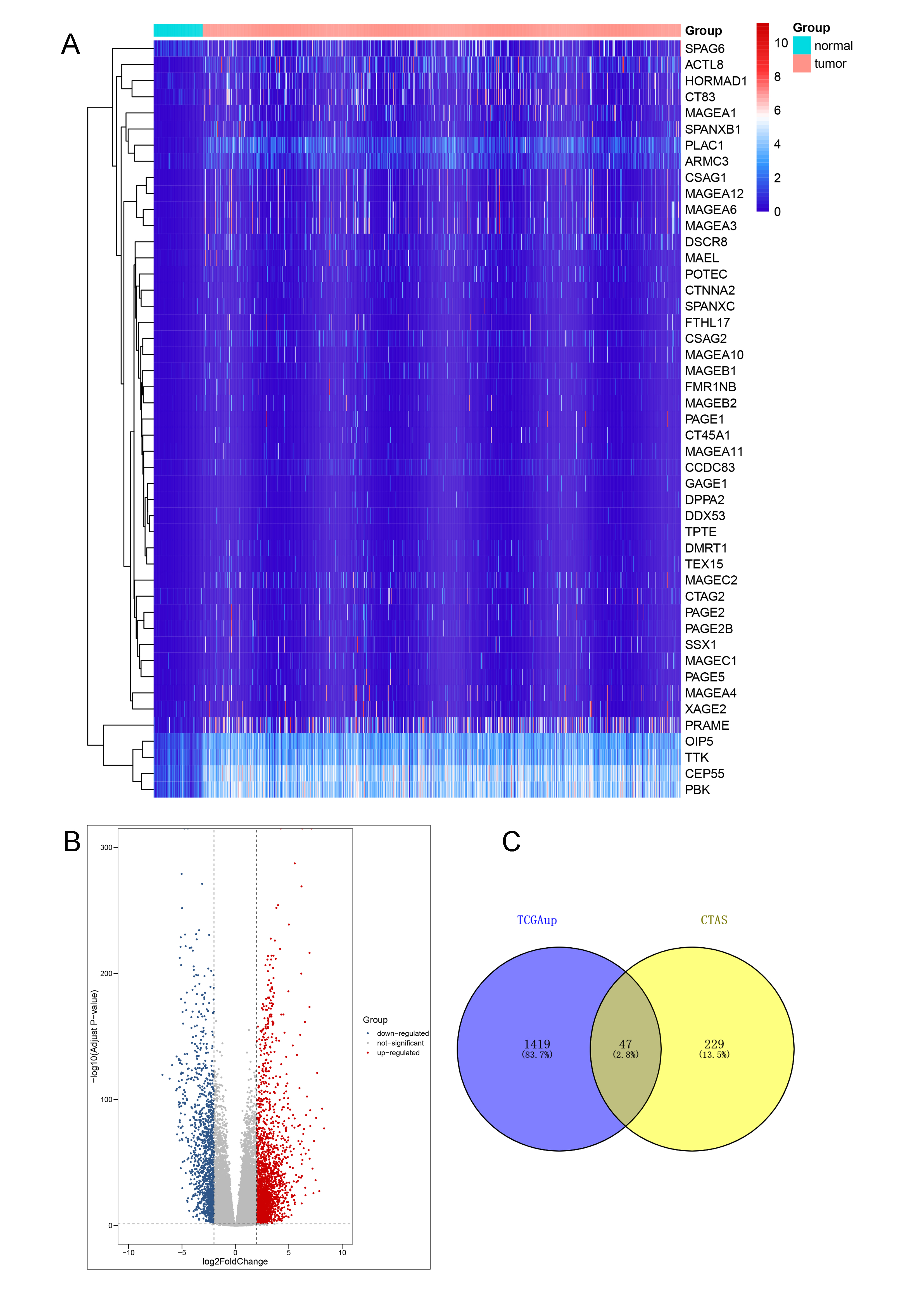

Supplement: Supplementary file 2 — Additional file 2. Expression of CTAs in TCGA breast cancer data. A: Heatmap of 47 CTAs in TCGA breast cancer data. B: Volcano plot of differentially expressed genes in breast cancer from TCGA database. C: Venn diagram of the intersection of 276 CTAs and upregulated genes in TCGA breast cancer data. [file 12967_2023_4030_MOESM2_ESM.jpg]

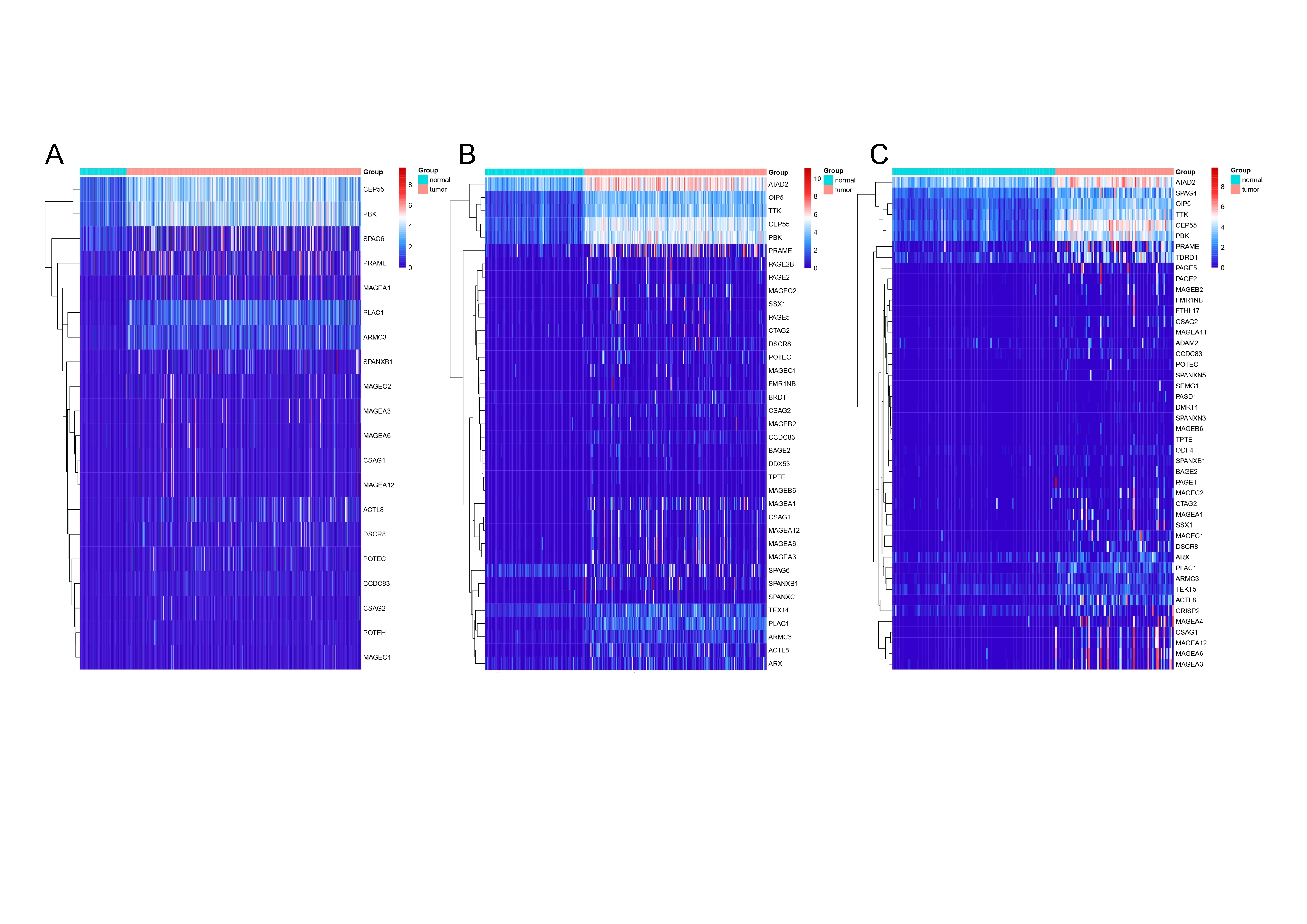

Supplement: Supplementary file 3 — Additional file 3. Expression of CTAs in TCGA luminal A, luminal B, and HER2 + breast cancer data. A: Heatmap of the expression of 20 CTAs in TCGA luminal A type breast cancer data. B: Heatmap of the expression of 37 CTAs in TCGA luminal B type breast cancer data. C: Heatmap of the expression of 46 CTAs in TCGA HER2 positive breast cancer data. [file 12967_2023_4030_MOESM3_ESM.jpg]

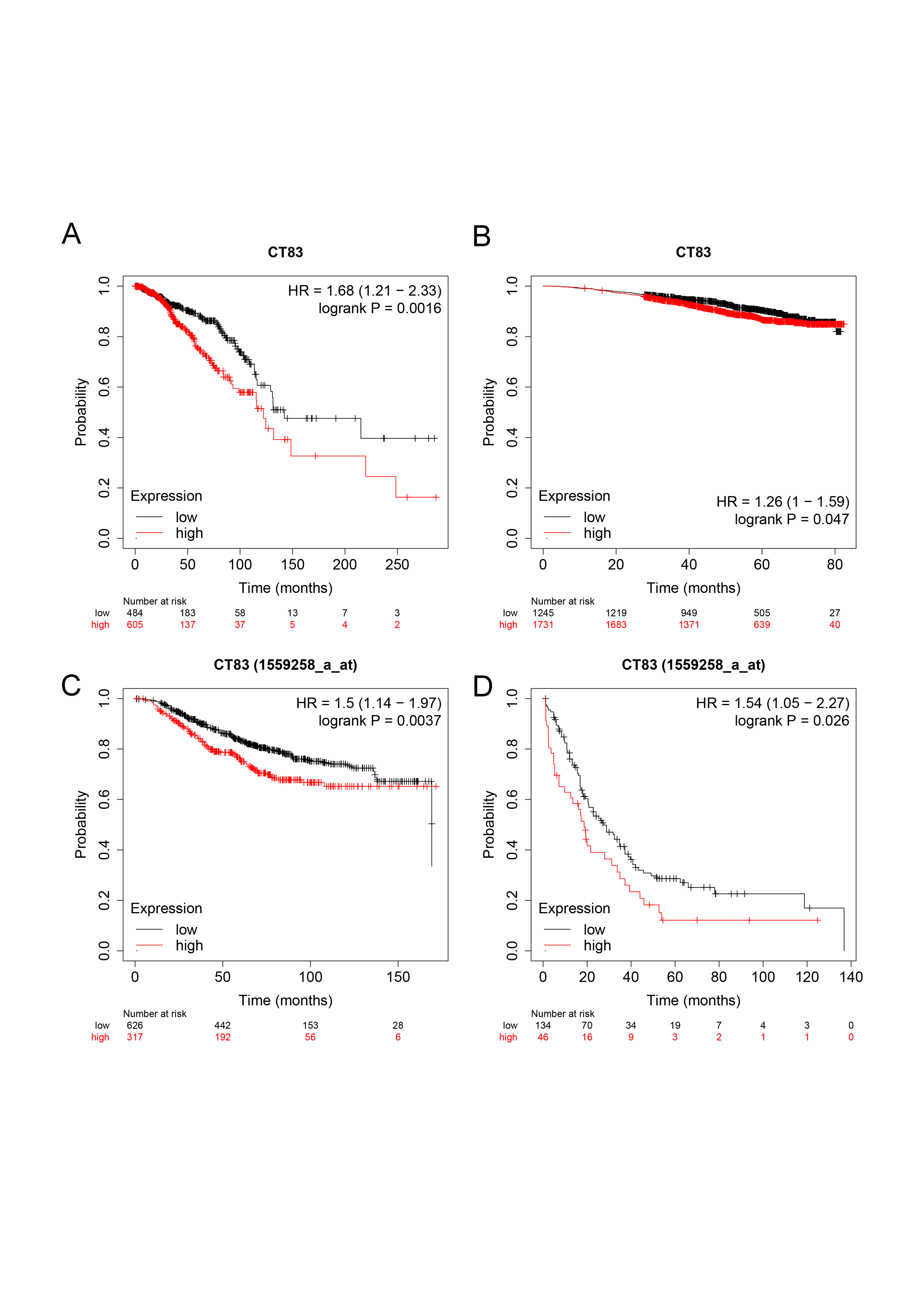

Supplement: Supplementary file 4 — Additional file 4. The role of KK-LC-1 expression in the prognosis of breast cancer patients using the Kaplan–Meier plotter database. A: The effect of increased KK-LC-1 mRNA expression on OS was analyzed in a study including 1089 patients. B: The effect of increased KK-LC-1 mRNA expression on OS was analyzed in a study including 2976 patients. C: The effect of increased KK-LC-1 mRNA expression on OS was analyzed in a study that included 943 patients. D: The effect of increased KK-LC-1 mRNA expression on PPS was analyzed in a study including 180 patients. [file 12967_2023_4030_MOESM4_ESM.jpg]

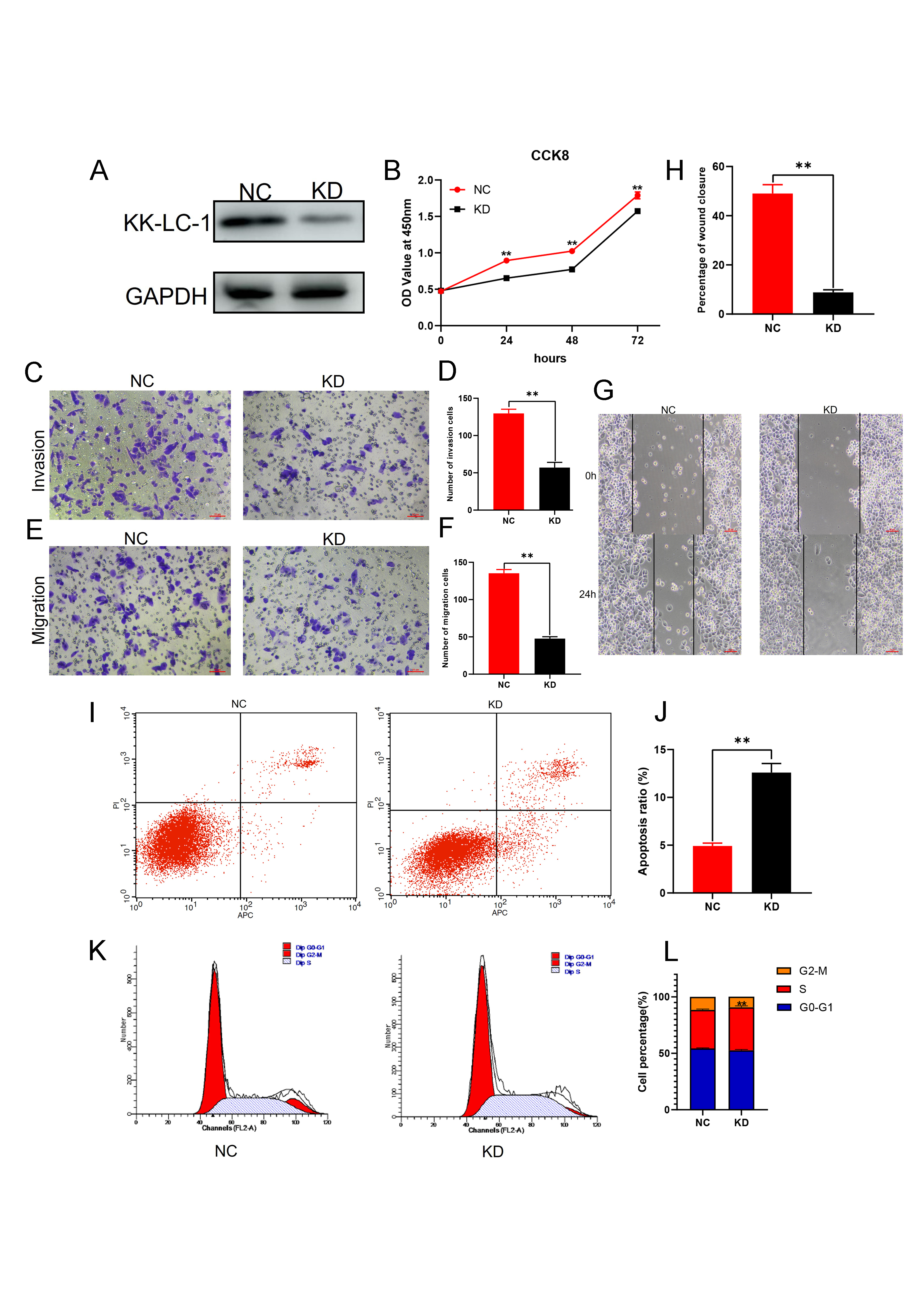

Supplement: Supplementary file 5 — Additional file 5. KK-LC-1 regulates the malignant biological behaviors of MDA-MB-468 triple-negative breast cancer cells. A: The expression of KK-LC-1 in MDA-MB-468 cells was silenced by lentivirus and verified by western blotting. B: Proliferation of MDA-MB-468/NC and MDA-MB-468/KD cells determined by the CCK8 assay. C-D: Invasive ability of MDA-MB-468/NC and MDA-MB-468/KD cells determined by the Transwell assay. E–F: Migration ability of MDA-MB-468/NC and MDA-MB-468/KD cells determined by the Transwell assay. G, H: Differential analysis of the scratch healing ability of MDA-MB-468/NC and MDA-MB-468/KD cells. I, J: Detection of apoptosis in MDA-MB-468/NC and MDA-MB-468/KD cells using flow cytometry. K, L: Cell cycle detection of MDA-MB-468/NC and MDA-MB-468/KD cells using flow cytometry. (*P < 0.05, **P < 0.01) [file 12967_2023_4030_MOESM5_ESM.jpg]

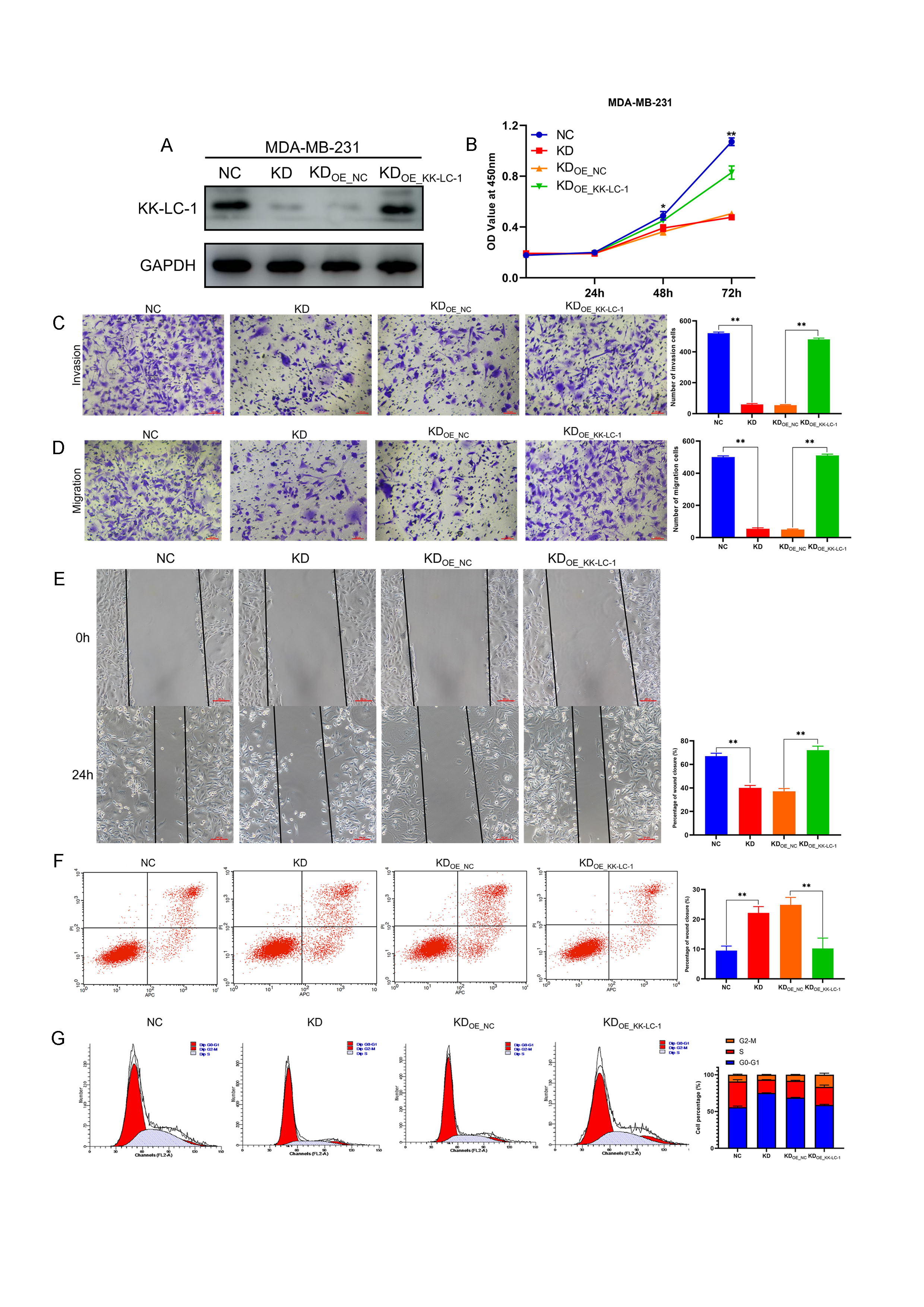

Supplement: Supplementary file 6 — Additional file 6. The effect of KK-LC-1 expression rescue on the malignant biological behaviors of MDA-MB-231 triple-negative breast cancer cells. A: Detection of KK-LC-1 expression in MDA-MB-231/NC, MDA-MB-231/KD, MDA-MB-231/KDOE_NC, and MDA-MB-231/KDOE_KK-LC-1 cells using western blotting. B: Proliferation of MDA-MB-231/NC, MDA-MB-231/KD, MDA-MB-231/KDOE_NC, and MDA-MB-231/KDOE_KK-LC-1 cells detected using the CCK8 assay. C: Transwell assay to detect the invasive ability of MDA-MB-231/NC, MDA-MB-231/KD, MDA-MB-231/KDOE_NC, and MDA-MB-231/KDOE_KK-LC-1 cells. D: Transwell assay to detect the migration ability of MDA-MB-231/NC, MDA-MB-231/KD, MDA-MB-231/KDOE_NC, and MDA-MB-231/KDOE_KK-LC-1 cells. E: Differential analysis of the scratch healing ability of MDA-MB-231/NC, MDA-MB-231/KD, MDA-MB-231/KDOE_NC, and MDA-MB-231/KDOE_KK-LC-1 cells. F: Detection of apoptosis in MDA-MB-231/NC, MDA-MB-231/KD, MDA-MB-231/KDOE_NC, and MDA-MB-231/KDOE_KK-LC-1 cells using flow cytometry. G: Cell cycle detection in MDA-MB-231/NC, MDA-MB-231/KD, MDA-MB-231/KDOE_NC, and MDA-MB-231/KDOE_KK-LC-1 cells using flow cytometry. MDA-MB-231/KDOE_NC cells: a group of empty plasmid-transfected KK-LC-1-silenced MDA-MB-231 cell lines. MDA-MB-231/KDOE_KK-LC-1 cells: a group of KK-LC-1 overexpression plasmid-transfected KK-LC-1-silenced MDA-MB-231 cell lines.(**P < 0.01). [file 12967_2023_4030_MOESM6_ESM.jpg]

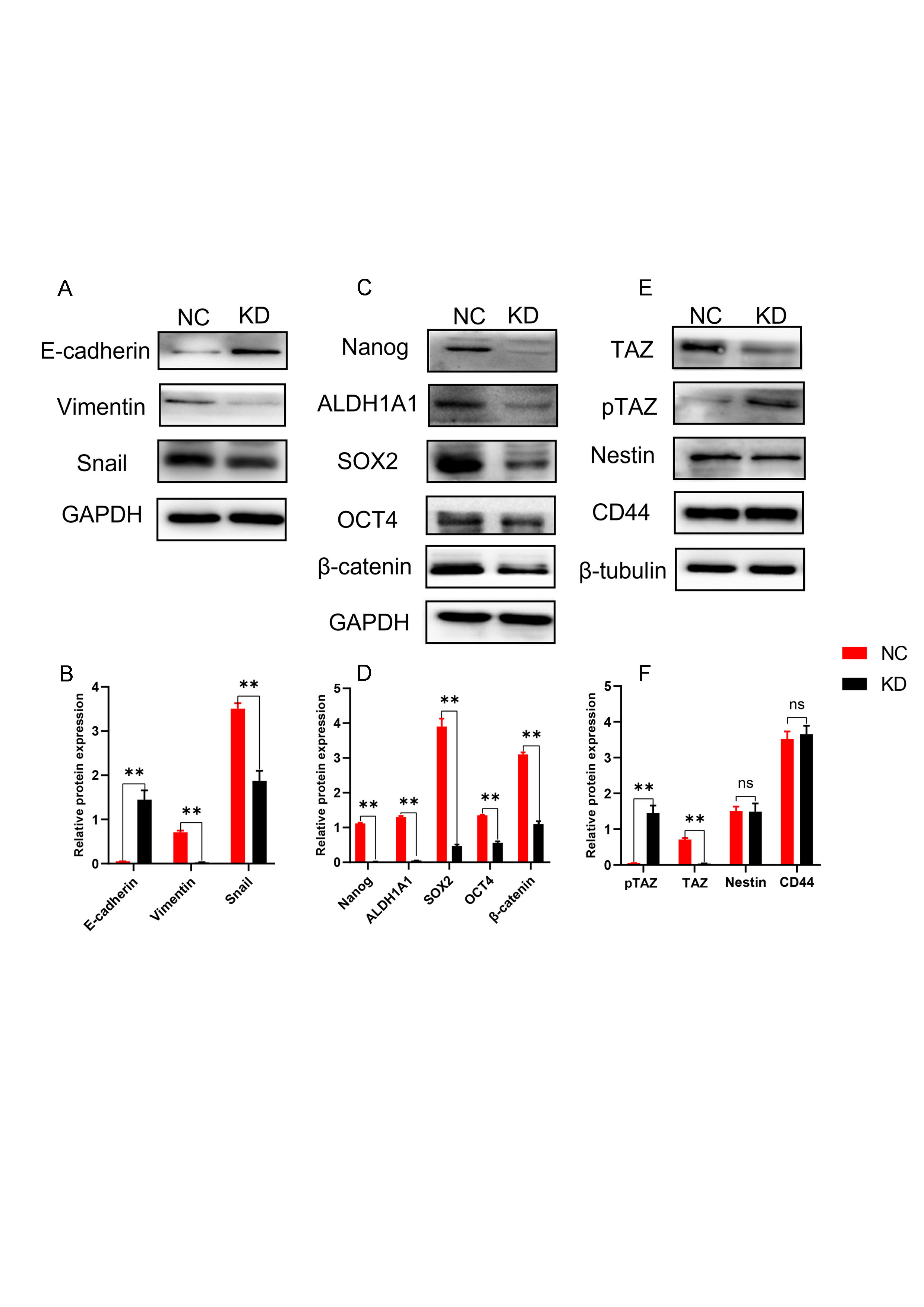

Supplement: Supplementary file 7 — Additional file 7. Effects of KK-LC-1 silencing on the expression of EMT and BCSCs markers. A, B: Expression of E-cadherin, vimentin, and Snail in MDA-MB-231/NC and MDA-MB-231/KD cells was detected using western blotting (**P < 0.01). C, D: Expression of Nanog, ALDH1A1, SOX2, OCT4, and β-catenin in MDA-MB-231/NC and MDA-MB-231/KD cells was detected using western blot (**P < 0.01). E, F: The expression of TAZ, pTAZ, Nestin and CD44 in MDA-MB-231/NC and MDA-MB-231/KD cells was detected using western blotting (**P < 0.01). [file 12967_2023_4030_MOESM7_ESM.jpg]

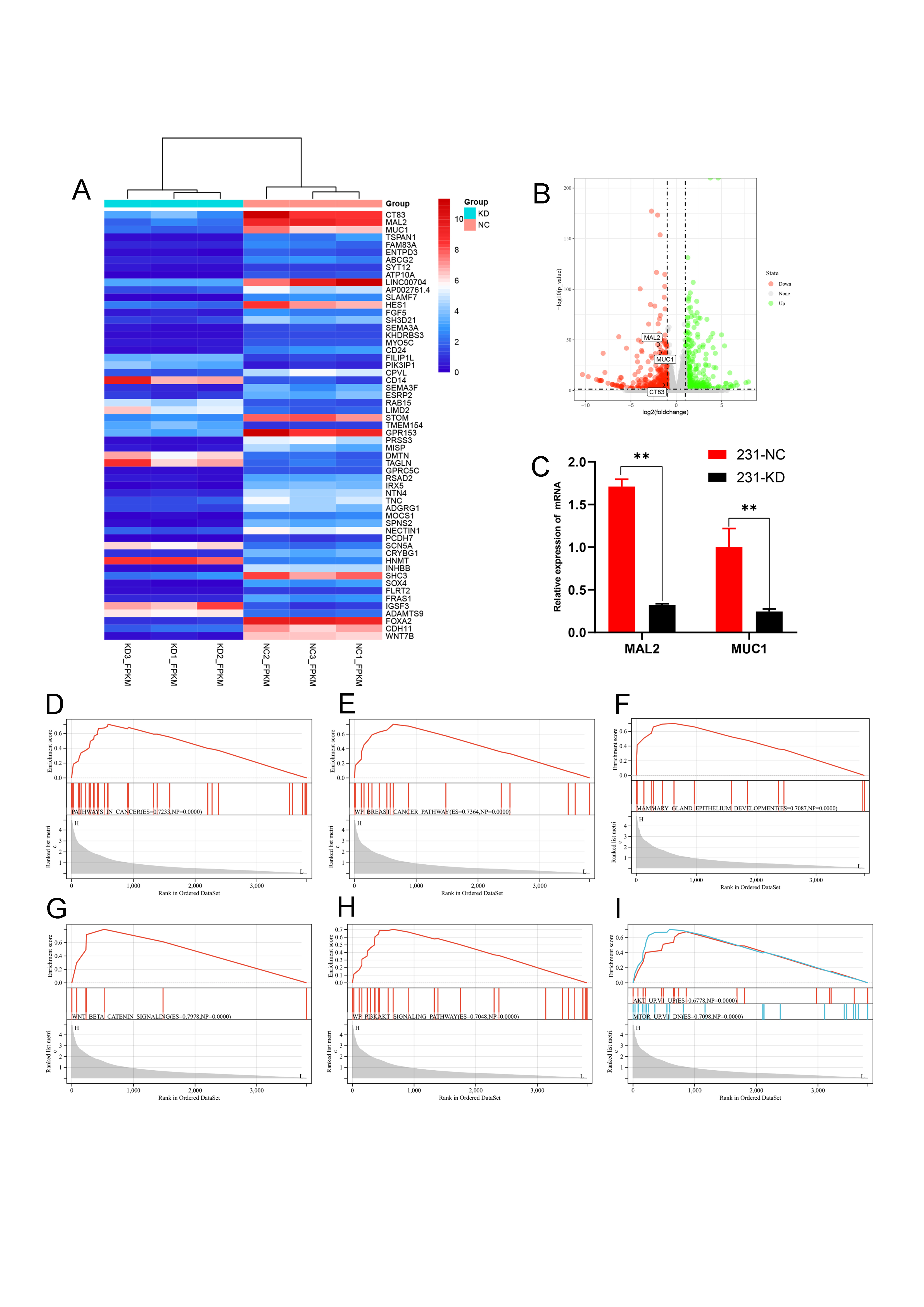

Supplement: Supplementary file 8 — Additional file 8. NGS results and GSEA analysis of MDA-MB-231/NC and MDA-MB-231/KD cells. A: Representative differential gene expression heatmap obtained using NGS. B: Volcano plot of differential gene expression using NGS. C: Validation the mRNA level of MAL2 and MUC1 in MDA-MB-231/NC and MDA-MB-231/KD cells by RT-qPCR. D-I: KK-LC-1 single-gene GSEA enrichment analysis using NGS data. [file 12967_2023_4030_MOESM8_ESM.jpg]

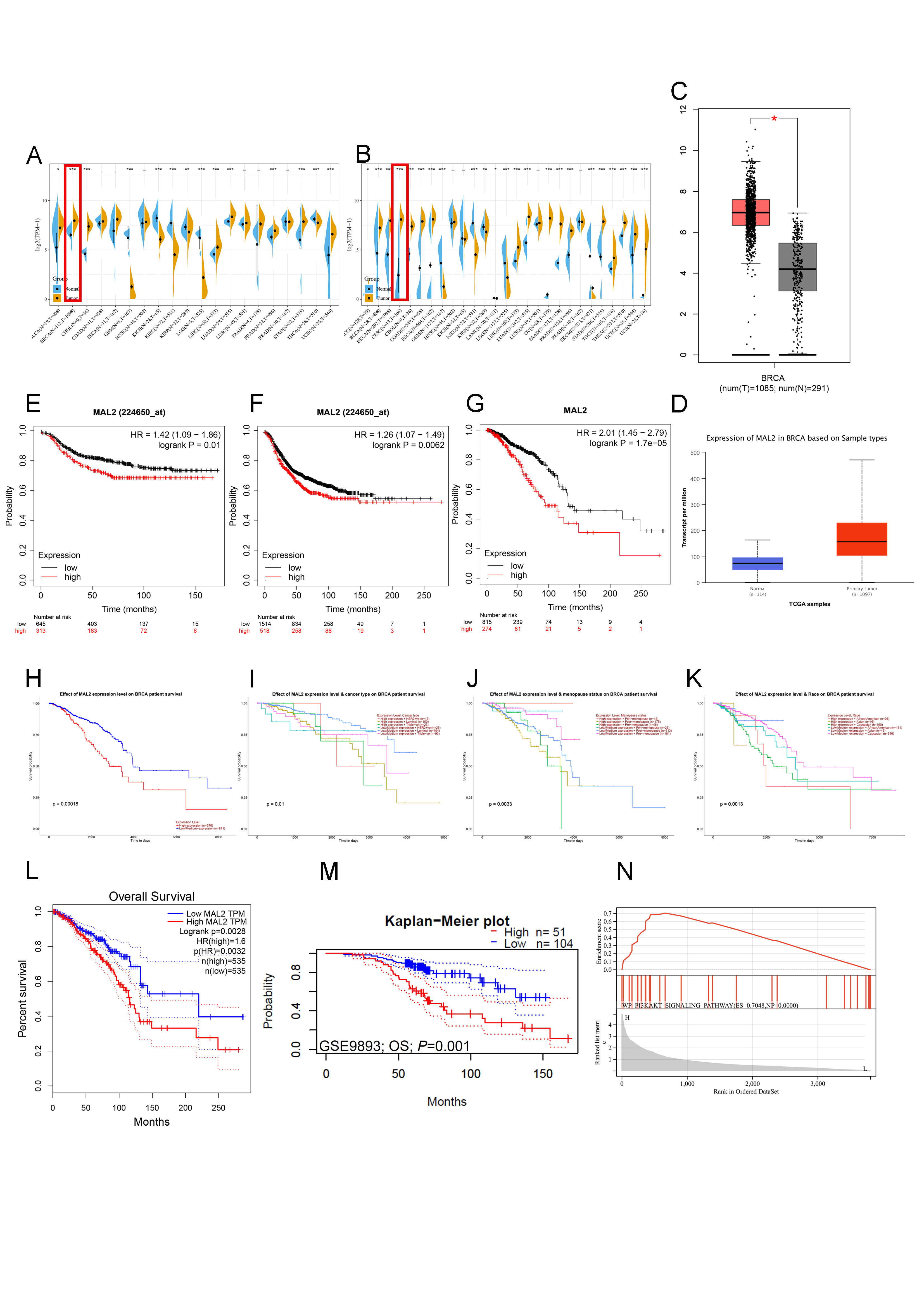

Supplement: Supplementary file 9 — Additional file 9. Bioinformatic methods integrated analysis of MAL2 expression and prognostic role in breast cancer, and GSEA analysis of MAL2 gene. A: The Sangerbox tool was used to explore the expression of MAL2 mRNA by analyzing the data from breast cancer and adjacent cancer tissues. B: The Sangerbox tool was used to explore the expression of MAL2 mRNA by further integrating the data from normal breast tissues in the GTEx database. C: Analysis of MAL2 mRNA expression in breast cancer and adjacent normal tissues using the GEPIA database. D: Analysis of MAL2 mRNA expression in breast cancer and adjacent normal tissues using the UALCAN database. E: Effects of high MAL2 mRNA expression on DFS in a study including 958 patients. F: The effect of high MAL2 mRNA expression on RFS in a study including 2032 patients. G: Effects of high MAL2 mRNA expression on OS in a study including 1089 patients. H: Effects of high MAL2 mRNA expression on the survival of patients with breast cancer was analyzed using the UALCAN database. I: Effects of high MAL2 mRNA expression on the survival of breast cancer patients with different molecular types, using the UALCAN database. J: Effects of high MAL2 mRNA expression on the survival of breast cancer patients with different menopausal statuses, using the UALCAN database. K: Effects of high MAL2 mRNA expression on the survival of breast cancer patients of different races, using the UALCAN database. L: Effects of high MAL2 mRNA expression on OS in breast cancer patients using the GEPIA database. M: Effects of high MAL2 mRNA expression on OS in patients with breast cancer in the GSE9893 database. N: GSEA analysis of MAL2 gene. [file 12967_2023_4030_MOESM9_ESM.jpg]

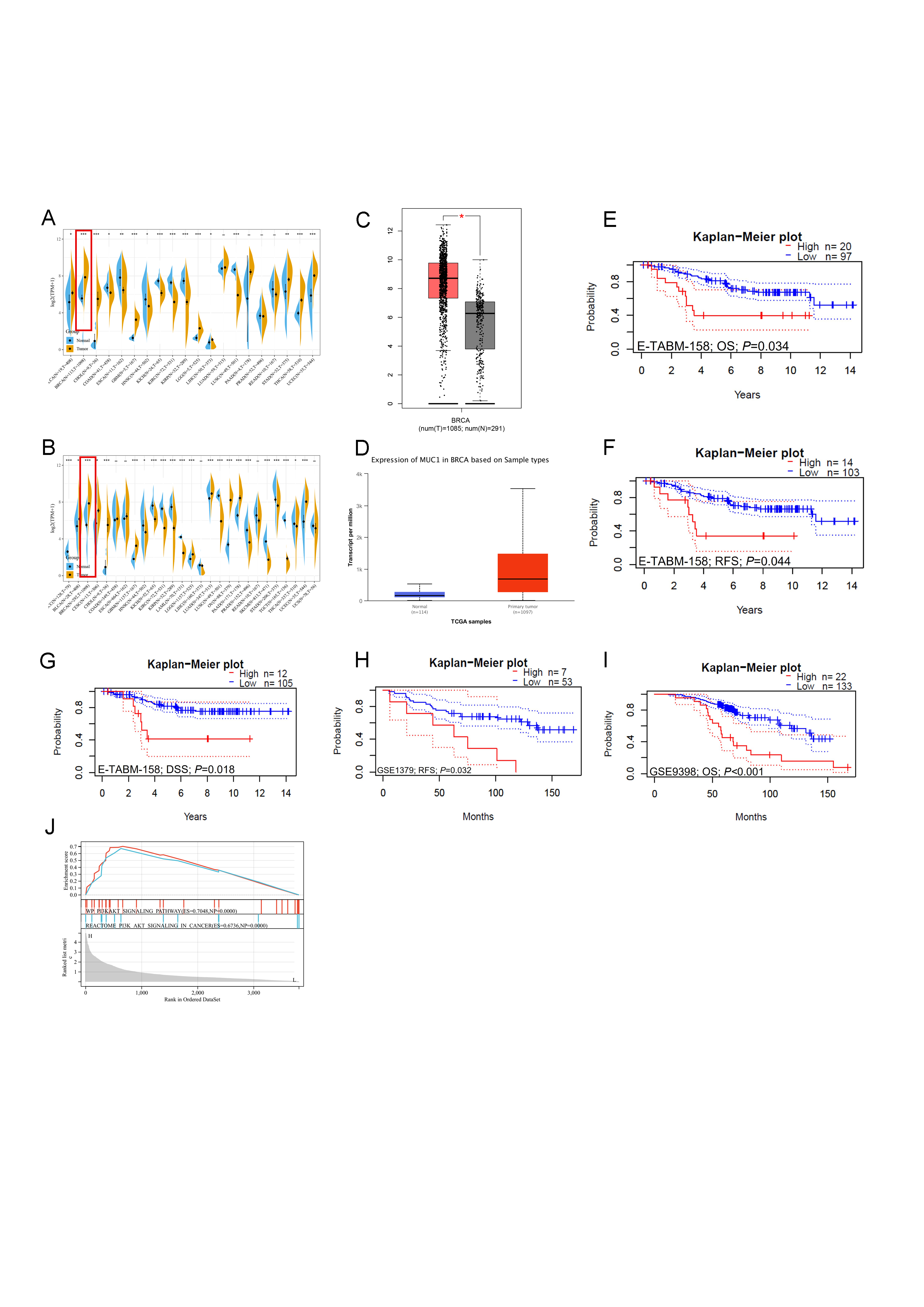

Supplement: Supplementary file 10 — Additional file 10. Bioinformatic methods integrated analysis of MUC1 expression and prognostic role in breast cancer, and GSEA analysis of MUC1 gene. A: The Sangerbox tool was used to explore the expression of MUC1 mRNA by analyzing the data from breast cancer and adjacent cancer tissues. B: The Sangerbox tool was used to explore the expression of MUC1 mRNA by further integrating the data from normal breast tissues in the GTEx database. C: Analysis of MUC1 mRNA expression in breast cancer and adjacent normal tissues using the GEPIA database. D: Analysis of MUC1 mRNA expression in breast cancer and adjacent normal tissues using the UALCAN database. E: Effects of high MUC1 mRNA expression on OS in the E-TABM-158 database. F: Effects of high MUC1 mRNA expression on RFS in the E-TABM-158 database. G: Effects of high MUC1 mRNA expression on DSS in the E-TABM-158 database. H: Effects of high MUC1 mRNA expression on RFS in the GSE1379 database. I: Effects of high MUC1 mRNA expression on OS in the GSE9398 database. J: GSEA analysis of MUC1 gene. [file 12967_2023_4030_MOESM10_ESM.jpg]

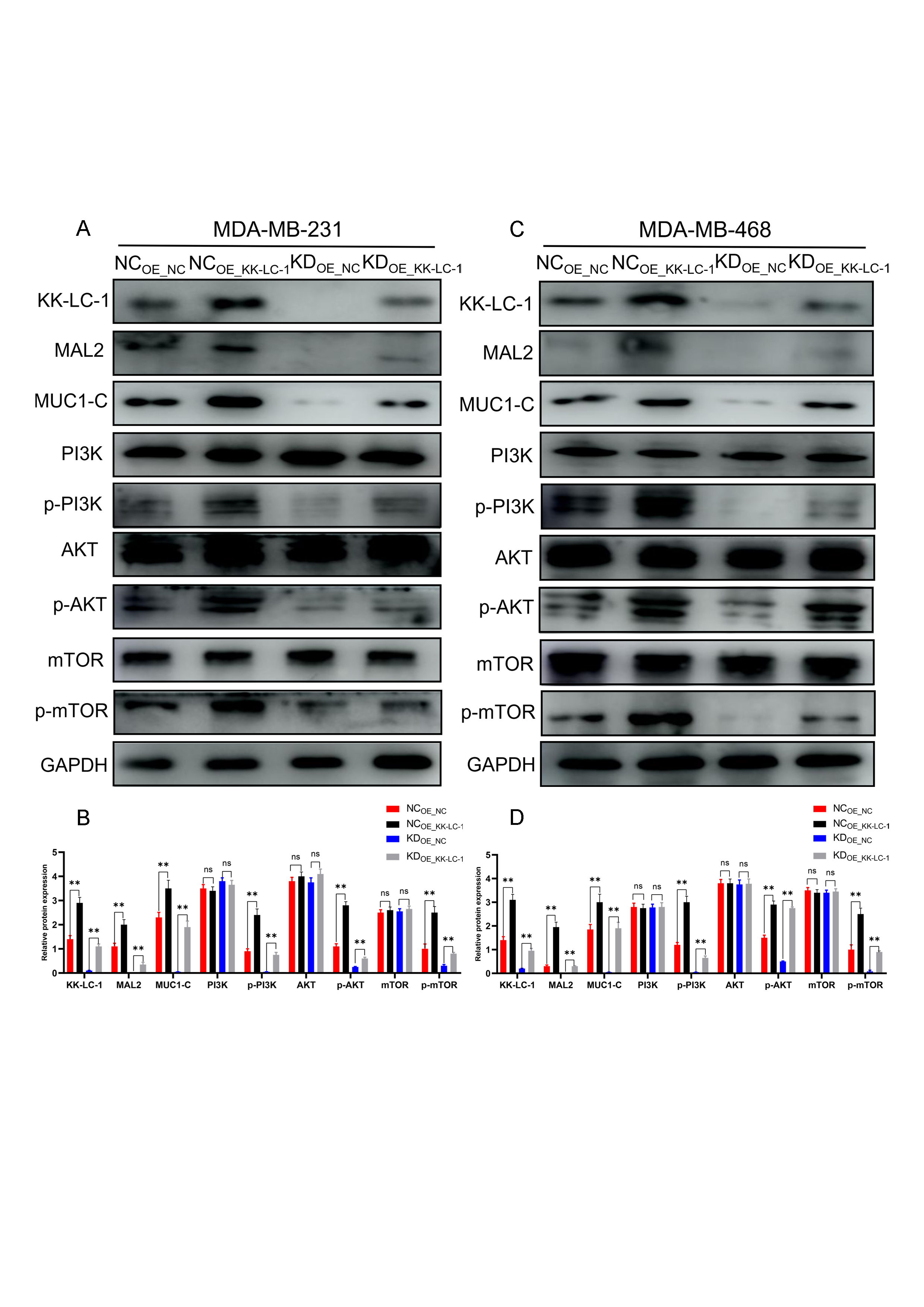

Supplement: Supplementary file 11 — Additional file 11. Rescue experiment to evaluate the effect of re-expressing KK-LC-1 in NC and KD cells on MAL2/MUC1-C/PI3K/AKT/mTOR pathway. A, B: Detection of KK-LC-1, MAL2, MUC1-C, PI3K, p-PI3K, AKT, p-AKT, mTOR, and p-mTOR expression in MDA-MB-231/NCOE_NC, MDA-MB-231/NCOE_KK-LC-1, MDA-MB-231/KDOE_NC and MDA-MB-231/KDOE_KK-LC-1 cells using western blotting (**P < 0.01). C, D: Detection of KK-LC-1, MAL2, MUC1-C, PI3K, p-PI3K, AKT, p-AKT, mTOR, and p-mTOR expression in MDA-MB-468/NCOE_NC, MDA-MB-468/NCOE_KK-LC-1, MDA-MB-468/KDOE_NC and MDA-MB-468/KDOE_KK-LC-1 cells using western blotting (**P < 0.01). NCOE_NC (The group of empty plasmid-transfected NC cell lines). NCOE_KK-LC-1 (The group of KK-LC-1 overexpression plasmid-transfected KD cell lines). KDOE_NC (The group of empty plasmid-transfected KD cell lines); KDOE_KK-LC-1 (The group of KK-LC-1 overexpression plasmid-transfected KD cell lines). [file 12967_2023_4030_MOESM11_ESM.jpg]

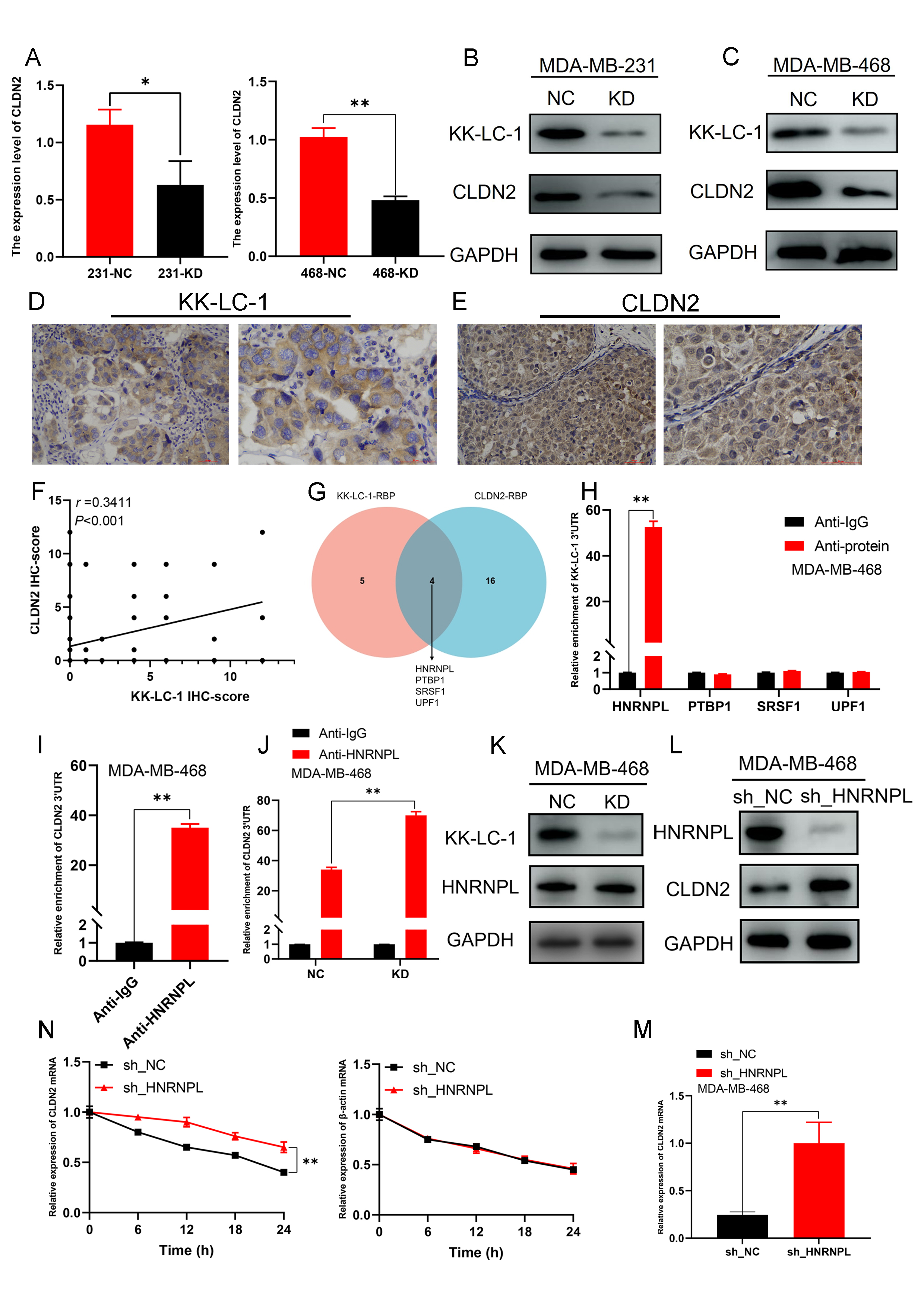

Supplement: Supplementary file 12 — Additional file 12: Fig. S15. KK-LC-1 may promote breast cancer liver metastasis by regulating the expression of CLDN2. A: Detection of CLDN2 mRNA expression in MDA-MB-231/NC, MDA-MB-231/KD MDA-MB-468/NC, MDA-MB-468/KD cells (*P < 0.05, **P < 0.01). B: Detection of CLDN2 protein expression in MDA-MB-231/NC and MDA-MB-231/KD cells using western blotting. C: Detection of CLDN2 protein expression in MDA-MB-468/NC and MDA-MB-468/KD cells using western blotting. D: Representative images of high KK-LC-1 protein expression (Left: 200× magnification. Right: 400× magnification). E: Representative images of high CLDN2 protein expression (Left: 200× magnification. Right: 400× magnification). F: Scatter plot of the correlation between KK-LC-1 and CLDN2 protein expression. G: The common RBPs of KK-LC-1 and CLDN2 were obtained through ENCORI. H: The binding between KK-LC-1 and candidate RBPs (HNRNPL, PTBP1, SRSF1 and UPF1) was assessed in MAD-MB-468 cells by RIP assay. (**P < 0.01). I: The binding between CLDN2 and HNRNPL was assessed in MAD-MB-468 cells by RIP assay. (**P < 0.01). J: The binding between CLDN2 and HNRNPL was analyzed by RIP assay with KK-LC-1 silencing. (**P < 0.01). K: Detection of KK-LC-1 and HNRNPL protein expression in MDA-MB-468/NC and MDA-MB-468/KD cells using western blotting. L: Detection of HNRNPL and CLDN2 protein expression in MDA-MB-468/sh_NC and MDA-MB-468/sh_HNRNPL cells using western blotting. M: Detection of CLDN2 mRNA expression in MDA-MB-468/sh_NC and MDA-MB-468/sh_HNRNPL cells using RT-qPCR. (**P < 0.01). N: The effect of HNRNPL on CLDN2 mRNA stability was assessed by RT-qPCR with the treatment of α-amanitin (an inhibitor of mRNA synthesis). [file 12967_2023_4030_MOESM12_ESM.jpg]

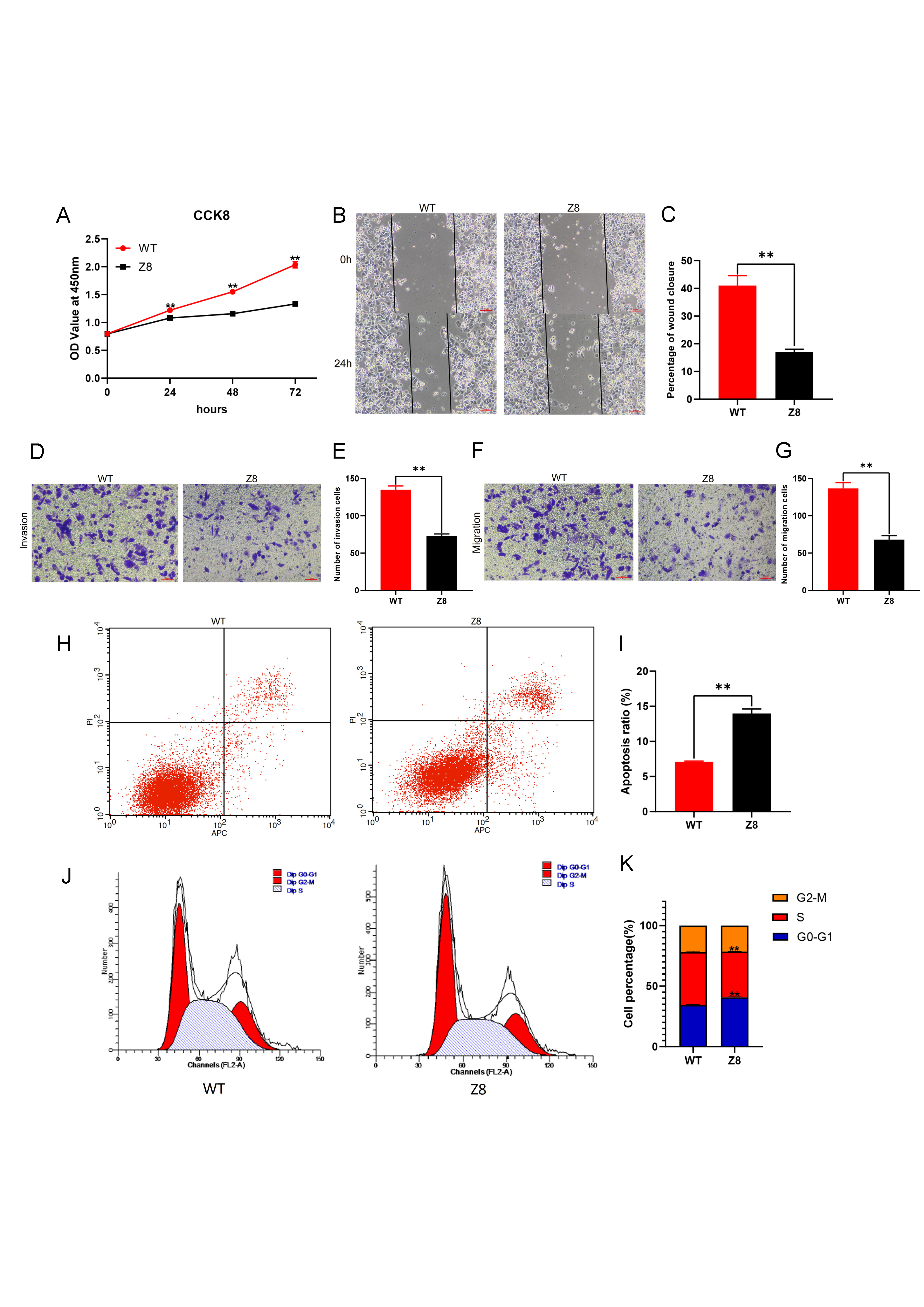

Supplement: Supplementary file 13 — Additional file 13. Inhibitory effect of Z8 small-molecule compound on malignant biological behaviors of MDA-MB-468 cells. A: Inhibitory effect of Z8 on the proliferation. B, C: Inhibitory effect of Z8 on the wound healing ability. D, E: Inhibitory effect of Z8 on the invasive ability. F, G: Inhibitory effect of Z8 on the migration ability. H, I: Z8 promotes apoptosis. J, K: Z8 blocks the cell cycle. (**P < 0.01). [file 12967_2023_4030_MOESM13_ESM.jpg]

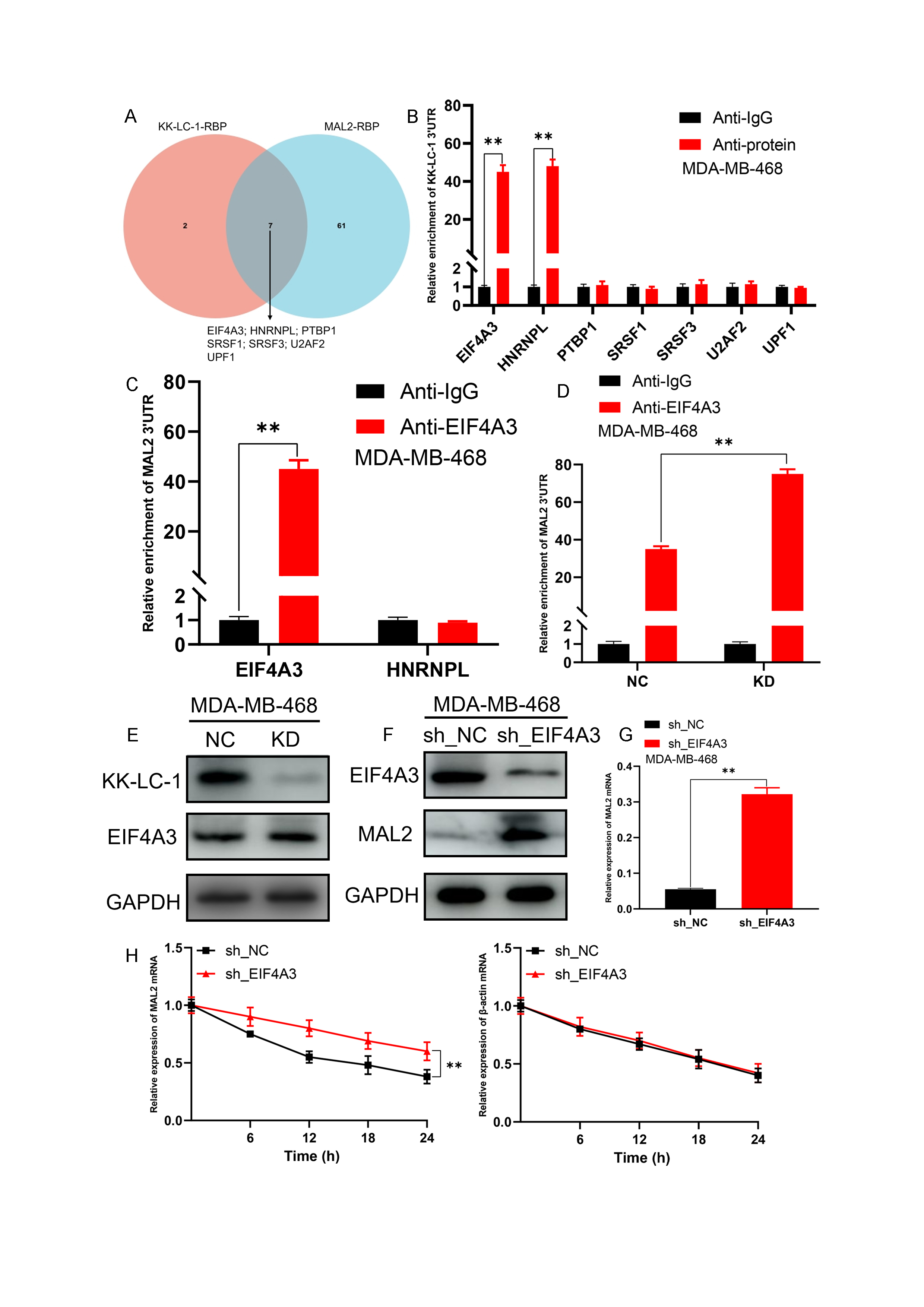

Supplement: Supplementary file 14 — Additional file 14. The molecular mechanism that KK-LC-1 regulated the expression of MAL2. A: The common RBPs of KK-LC-1 and MAL2 were obtained through ENCORI. B: The binding between KK-LC-1 and candidate RBPs (EIF4A3, HNRNPL, PTBP1, SRSF1, SRSF3, U2AF2 and UPF1) was assessed in MAD-MB-468 cells by RIP assay. (**P < 0.01). C: The binding between MAL2 and EIF4A3, HNRNPL was assessed in MAD-MB-468 cells by RIP assay. (**P < 0.01). D: The binding between MAL2 and EIF4A3 was analyzed by RIP assay with KK-LC-1 silencing. (**P < 0.01). E: Detection of KK-LC-1 and EIF4A3 protein expression in MDA-MB-468/NC and MDA-MB-468/KD cells using western blotting. F: Detection of EIF4A3 and MAL2 protein expression in MDA-MB-468/sh_NC and MDA-MB-468/sh_EIF4A3 cells using western blotting. G: Detection of MAL2 mRNA expression in MDA-MB-468/sh_NC and MDA-MB-468/sh_EIF4A3 cells using RT-qPCR. (**P < 0.01). H: The effect of EIF4A3 on MAL2 mRNA stability was assessed by RT-qPCR with the treatment of α-amanitin (an inhibitor of mRNA synthesis). [file 12967_2023_4030_MOESM14_ESM.jpg]

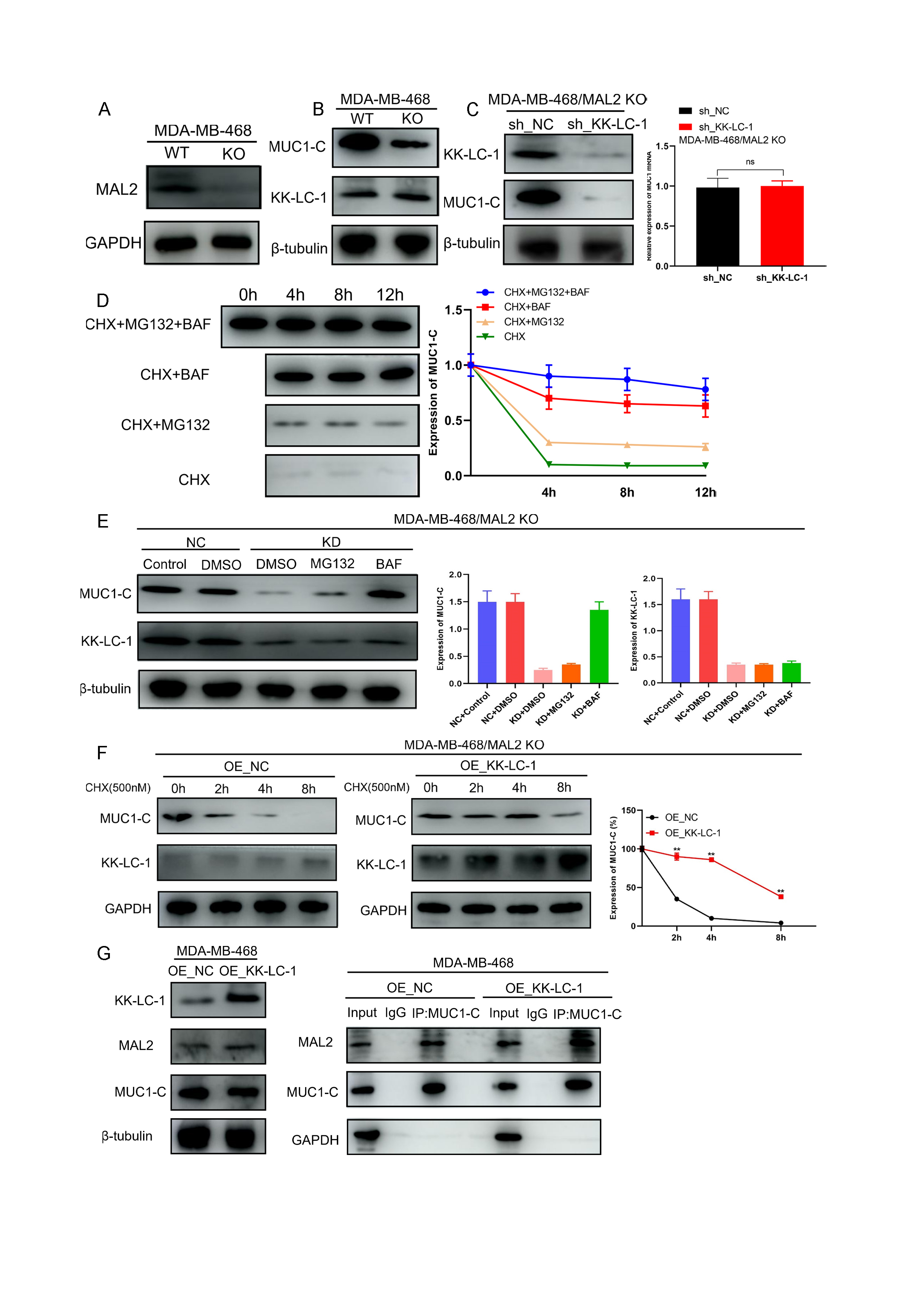

Supplement: Supplementary file 15 — Additional file 15. The molecular mechanism that KK-LC-1 directly regulated the expression of MUC1-C. A: Western blotting showing MAL2 protein levels in MA-MB-468/WT and MDA-MB-468/KO cells. MDA-MB-468/KO cells: MAL2 expression was knocked out by CRISPR-Cas9 in MDA-MB-468 cells. B: Western blotting showing MUC1-C and KK-LC-1 protein levels in MA-MB-468/WT and MDA-MB-468/KO cells. C: Western blotting showing KK-LC-1 and MUC1-C protein levels in KK-LC-1 silenced MDA-MB-468/KO cells. D: Western blotting showing MUC1-C protein levels in MDA-MB-468/KO cells treated with different combinations of cycloheximide, MG132 and bafilomycin at different time points. E: Western blotting showing MUC1-C protein levels in KK-LC-1 silenced MDA-MB-468/KO cells treated with MG132 and bafilomycin. F: Western blotting showing degradation of MUC1-C protein in KK-LC-1 overexpressed MDA-MB-468/KO cells and control MDA-MB-468/KO cells. G: Western blotting showing the expression of KK-LC-1, MAL2 and MUC1-C protein in KK-LC-1 overexpressed MDA-MB-468/KO cells and control MDA-MB-468/KO cells with the treatment of CHX. And immunoprecipitation assay showed that KK-LC-1 overexpression can promote the interaction between MAL2 and MUC1-C. OE_NC (The group of empty plasmid-transfected MDA-MB-468/KO cells). OE_KK-LC-1 (The group of KK-LC-1 overexpression plasmid-transfected MDA-MB-468/KO cells) [file 12967_2023_4030_MOESM15_ESM.jpg]
